# Convolutional Neural Net-Based Cassava Storage Root Counting Using Real and Synthetic Images

**DOI:** 10.3389/fpls.2019.01516

**Published:** 2019-11-26

**Authors:** John Atanbori, Maria Elker Montoya-P, Michael Gomez Selvaraj, Andrew P. French, Tony P. Pridmore

**Affiliations:** ^1^Agrobiodiversity Research Area, School of Computer Science, University of Nottingham, Nottingham, United Kingdom; ^2^Agrobiodiversity Research Area, International Center for Tropical Agriculture (CIAT), Cali, Colombia

**Keywords:** convolutional neural networks, generative adversarial networks, cassava phenotyping, machine learning, root counting

## Abstract

Cassava roots are complex structures comprising several distinct types of root. The number and size of the storage roots are two potential phenotypic traits reflecting crop yield and quality. Counting and measuring the size of cassava storage roots are usually done manually, or semi-automatically by first segmenting cassava root images. However, occlusion of both storage and fibrous roots makes the process both time-consuming and error-prone. While Convolutional Neural Nets have shown performance above the state-of-the-art in many image processing and analysis tasks, there are currently a limited number of Convolutional Neural Net-based methods for counting plant features. This is due to the limited availability of data, annotated by expert plant biologists, which represents all possible measurement outcomes. Existing works in this area either learn a direct image-to-count regressor model by regressing to a count value, or perform a count after segmenting the image. We, however, address the problem using a direct image-to-count prediction model. This is made possible by generating synthetic images, using a conditional Generative Adversarial Network (GAN), to provide training data for missing classes. We automatically form cassava storage root masks for any missing classes using existing ground-truth masks, and input them as a condition to our GAN model to generate synthetic root images. We combine the resulting synthetic images with real images to learn a direct image-to-count prediction model capable of counting the number of storage roots in real cassava images taken from a low cost aeroponic growth system. These models are used to develop a system that counts cassava storage roots in real images. Our system first predicts age group ('young' and 'old' roots; pertinent to our image capture regime) in a given image, and then, based on this prediction, selects an appropriate model to predict the number of storage roots. We achieve 91% accuracy on predicting ages of storage roots, and 86% and 71% overall percentage agreement on counting 'old' and 'young' storage roots respectively. Thus we are able to demonstrate that synthetically generated cassava root images can be used to supplement missing root classes, turning the counting problem into a direct image-to-count prediction task.

## Introduction

The tropical root crop, cassava (*Manihot esculenta Crantz*), is a staple food for more than a tenth of the world's population. However, a major obstacle reducing its industrial potential is it's long and variable growth cycle. Information on the development of the edible cassava storage root is therefore crucial for selecting high yielding, early bulking cassava root crops for industrial-scale production. Cassava root systems comprise two key types of root. Fibrous roots develop first, and only a small number of these go on to form the larger storage roots. It is these storage roots which become an important food source, in particular a major source of carbohydrates. Understanding the growth of these storage roots therefore becomes an important phenotyping task. Presently, phenotyping of cassava storage roots is carried out using manual, destructive sampling methods (Okogbenin et al., 2013; Belalcazar et al., 2016), which are labour-intensive and require many replications of each genotype. The physiological traits of the cassava crop are usually measured manually, often during harvesting, but also pre-harvest. Measurements begin in the third month and continue every month until harvested (Okogbenin et al., 2013). The important pre-harvest traits measured include the number of storage roots and the primary stems, while harvest traits include the above-ground biomass, stem diameter and number of storage roots, along with their length and volume."

Image-based software tool development and usage for plant phenotyping tasks have increased in recent years (Furbank and Tester, 2011). Ideally, such tools should be high-throughput and at least semi-automatic, making them capable of providing accurate, quantitative data on plant structure and function with minimal manual labour. Most current phenotyping installations require precisely-designed, automated image acquisition hardware matched to specialist software solutions to achieve the best quality data and throughput. Often, the function of the image analysis step is impeded if the images are not captured in a tightly controlled, systematic way. Nevertheless, these tools are gaining more attention due to their merits in providing large-scale plant phenotyping when compared with manual methods. Image-based phenotyping techniques have recently been used in plant segmentation (Aich and Stavness, 2017; Aich et al., 2018), leaf counting (Giuffrida et al., 2015; Aich et al., 2018; Aich and Stavness, 2017) and to automatically identify root and leaf tips (Pound et al., 2017a). Dedicated development frameworks are even available to make building custom systems easier. For example, PlantCV (Gehan et al., 2017) can support a number of plant phenotyping tasks *via* processing pipelines, and the Deep Plant Phenomics platform (Ubbens and Stavness, 2017) specifically supports deep learning development.

Recently, very deep Convolutional Neural Networks (CNNs) have been used to recover plant traits in an attempt to gain improved robustness and accuracy (Scharr et al., 2016; Minervini et al., 2016a; Minervini et al., 2016b; Aich and Stavness 2017; Pound et al., 2017b). Here, and in the broader computer vision community, these techniques have increased the accuracy of the image analysis, but require large numbers of data samples to make them sufficiently general. Deep networks can comprise very many parameters (in the millions), which in turn introduces expensive computations (Long et al., 2015; Yu and Koltun, 2015; Badrinarayanan et al., 2017; Lin et al., 2017; Zhao et al., 2017) and can make such models inefficient on low-cost, resource-limited devices.

To date, deep learning methods addressing feature counting tasks have focused almost exclusively on phenotyping the plant shoot system. Two broad approaches are in use. The first begins by segmenting the input image. Learning to segment requires individual annotation of the relevant objects to create a training data set, a task which is usually error prone and time-consuming for the plant biologist to undertake. The second approach learns a direct regression model. The regression approach solves this problem by using the total object count as its only supervision information, which is comparatively very easy to collect. A complete, pixel-by-pixel labeling of the training images is not required, only instead requiring a numerical label giving the count of the features of interest; e.g. a root count. The regression models which must be learned are, however, non-linear and of very high dimensionality (Aich and Stavness, 2017): Here, instead, we propose to develop a direct image-to-count prediction model instead.

**Figure 1:**
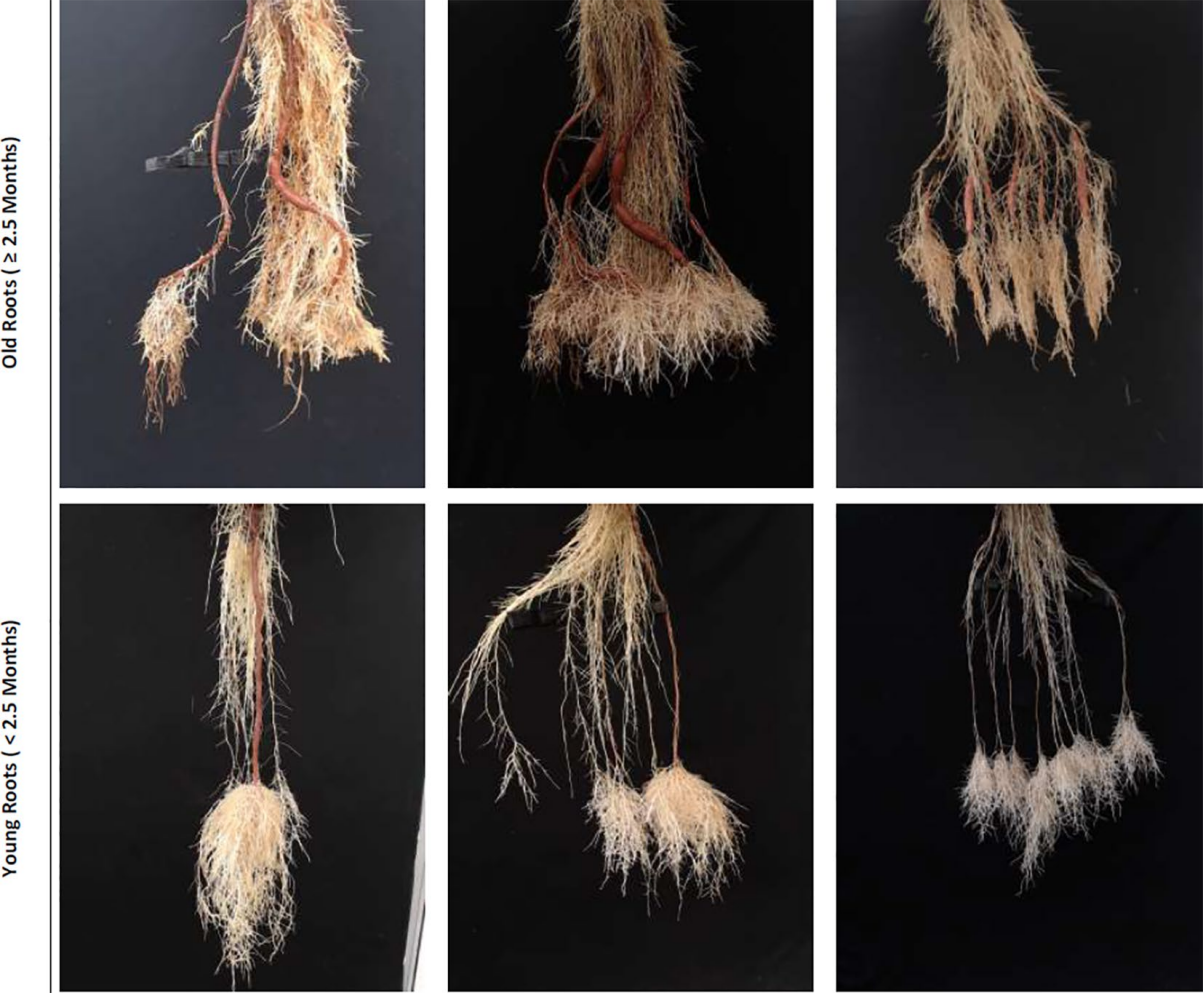
Sample real cassava root images from our datasets. The top row illustrates cassava roots that are 2.5 months and more, which we term “old” roots. The bottom row illustrates “young” roots, those less than 2.5 months.

We aim to develop a fully-automated, image-based phenotyping system to count storage roots in color images of aeroponically grown cassava, including and in particular counting early bulking storage roots (those appearing in the first 2.5 months of growth). The challenge here is that early storage roots are usually particularly difficult to detect. There are comparatively few such roots on any given plant, and they are often occluded by fibrous roots, which have similar color and texture (see example images in **Figure 1**, bottom row). We develop here a direct "image-to-count" prediction model, avoiding the complexity of the regression approach, and avoiding a pure segmentation approach which can be problematic when the boundaries of the objects involved (especially fibrous roots) are not well defined. This prediction approach effectively *classifies* each image according to the number of plant features, e.g. storage roots, present. This raises a further challenge: to successfully train a classifier a training set containing multiple images of each class is required. In the current context this means that we require images showing 0, 1, 2, 3, 4 etc, storage roots, up to the maximum number expected to be encountered. Though the number of roots that can be reasonably expected is not large—we do not need examples for every integer, of course—complete data sets of this type are often unavailable in existing repositories, and can take a significant amount of time to assemble. This is particularly true of more recently-studied species like cassava, for which limited image data exists. To remedy the problem of classes short on, or missing data, we first develop a conditional GAN to generate synthetic images of the storage roots classes which are not sufficiently well-represented with real image data.

The contributions of this work, then, are:

1. Design of a conditional Generative Adversarial Net (GAN) that can automatically generate synthetic cassava root images when presented with ground truth segmentation masks of the desired image classes.
2. Design of a deep CNN age-prediction model that predicts the age of cassava roots as either "old" (≥2.5 months) or "young" (≤2.5 months).
3. Design of a deep CNN-based storage root counting model, which given an input image and an age class will classify the image according to the number of storage roots present.
4. Combination of these components, which will create a cassava root counting tool to support an aeroponic phenotyping system (Selvaraj, 2019); this will be evaluated against a segmentation-based counting approach

The remainder of this paper is structured as follows. In *Background*, we review existing works that generate synthetic images using a GAN approach, and those that perform object counting using deep CNN systems. In *Datasets*, we introduce the cassava root datasets used in our experiments and proceed in *Image Prediction and Generation Methods* to describe our methods. We describe our experimental set-up, including a benchmark, in *Experimental Evaluation*. We then proceed to present and discuss our results in *Results* and draw conclusions in *Conclusion*.

## Background

As many current phenotyping techniques were initially developed in Europe and North America, where cereal crops dominate, comparatively few studies have phenotyped cassava (Subere et al., 2009; Okogbenin et al., 2013; Adu et al., 2018 (Polthanee et al., 2016). Though these studies have considered both shoot and root phenotyping, and some even examined roots regularly during their development (Okogbenin et al., 2013), measurement of cassava root traits is typically carried out only during harvest. Despite consideration of root numbers, size and length alongside shoot structural measures and biomass (Okogbenin et al., 2013; Adu et al., 2018), it has not yet been established which traits or variables provide the most detailed differentiation between distinctive genotypes (Adu et al., 2018).

Traditionally, cassava storage roots are phenotyped destructively using manual or semi-automated methods. This usually involves extracting the roots from the soil, losing a large number of small and fibrous roots in the process. Cassava roots are cut from their stem. They are then suspended in water to measure volume and spread on a black background to ease counting and measurement of their total length. Care is taken to avoid roots overlaying each other, as much as is practically possible. In semi-automated methods, a digital camera is used to capture root images. The controlled imaging conditions often allow simple thresholding methods to segment roots from background, and simple image analysis methods, controlled by the user, can be used to extract total root lengths and counts from the resulting binary images. This process is not only destructive to the plants but also time-consuming for the scientist.

Though there is existing literature presenting methods that *count* the plant features visible in an image, to our knowledge no study has considered automatically counting cassava storage roots. Automated counting methods have focussed on counting shoot features such as leaves (Giuffrida et al, 2015; Aich and Stavness, 2017; Ubbens and Stavness, 2017), plants (Ribera et al., 2017; Aich et al., 2018), seeds (Uzal et al., 2018), and fruits (Chen et al., 2017; Rahnemoonfar and Sheppard, 2017). Current approaches are all based on deep CNNs, and can be broadly divided into three categories: counting by segmentation, and direct image-to-count by class prediction or regression.

Methods that adopt a segmentation-based approach (Aich and Stavness, 2017; Aich et al., 2018) first identify pixels arising from the relevant plant component(s) in, usually, RGB images, using a CNN-based segmentation model. Aich et al. (Aich et al., 2018) counted leaves by summing the predictions of image patches from a deep CNN model. However, in Aich and Stavness, 2017, both the RGB image and the corresponding binary segmentation image produced by the CNN were used to estimate the number of leaves. The complexity of the images involved means that segmentation approaches may generate spurious segmentations that in turn lead to inaccurate counts.

Regression-based approaches usually pose the counting task as a non-linear regression problem, regressing the output of the final CNN to a single value which represents the object count. Giuffrida et al. (2015) used this approach, converting images to log-polar form to benefit from the information present in the natural radial structure of the plants. They extracted patches from the log-polar image to form a feature vector which was used to train a support vector regression network to predict leaf number. This study, however, uses perfect segmentation together with the image and it is not clear how robust the system is to segmentation errors.

Such a regression-based approach is effective, but introduces a non-linear regression problem of very high dimensionality, which can be avoided by image-to-count class prediction methods (Ubbens and Stavness, 2017; Ribera et al., 2017; Uzal et al., 2018). This approach treats the counting problem as one of classification. Direct image-to-count prediction methods that use a deep CNN typically have their final layer made up of a number of neurons equal to the maximum number of plant features to be counted. Ubbens and Stavness (Ubbens and Stavness, 2017) have shown that this method outperforms both the segmentation- and regression-based approaches. However, the problem with this approach is that samples representing all classes must be available. If some classes are not represented well, the network cannot be trained.

One way of overcoming this problem of missing data is to generate synthetic images for non-represented or under-represented classes. Various methods for generating synthetic data have been proposed in the computer vision literature. Recent state-of-the-art methods commonly use conditional GANs (Isola et al., 2017). A conditional GAN is a general-purpose solution to image-to-image translation problems. Conditional GANs learn the mapping from an input image to an output image, as well as a loss function to learn this mapping. Some previous works (Giuffrida et al., 2017; Ward et al., 2018; Zhu et al., 2018), have reported using a GAN for image-to-image mapping in plant phenotyping. These methods used the GAN to generate synthetic images to augment real data for leaf counting in the CVPPP 2017 LCC dataset (Scharr et al., 2017) and showed that the testing error is reduced compared with all the other state-of-the-art methods reported to date for the challenge.

## Datasets

We use two combined plant image datasets, which we refer to as the "old" and "young" cassava root sets, to perform all of our experiments. **Figure 1** shows samples taken from each of these. The top row comprises sample images drawn from the "old" dataset and the bottom from the "young." The "old" roots dataset is made up of cassava roots that are at least two and a half months old, while the young dataset contains images of roots that are less than two and a half months. Though they may have very similar size, color, and texture, some roots considered to be storage roots when seen in "young" cassava plants would not be classed as storage roots when they appear later in the plant's development, in "old" plants: storage root identification must therefore consider plant age. We therefore first classify an unseen image as containing "new" or "old" roots, then count storage roots taking that classification into account, as the analysis challenge presented by the two age groups is very different.

**Figure 2:**
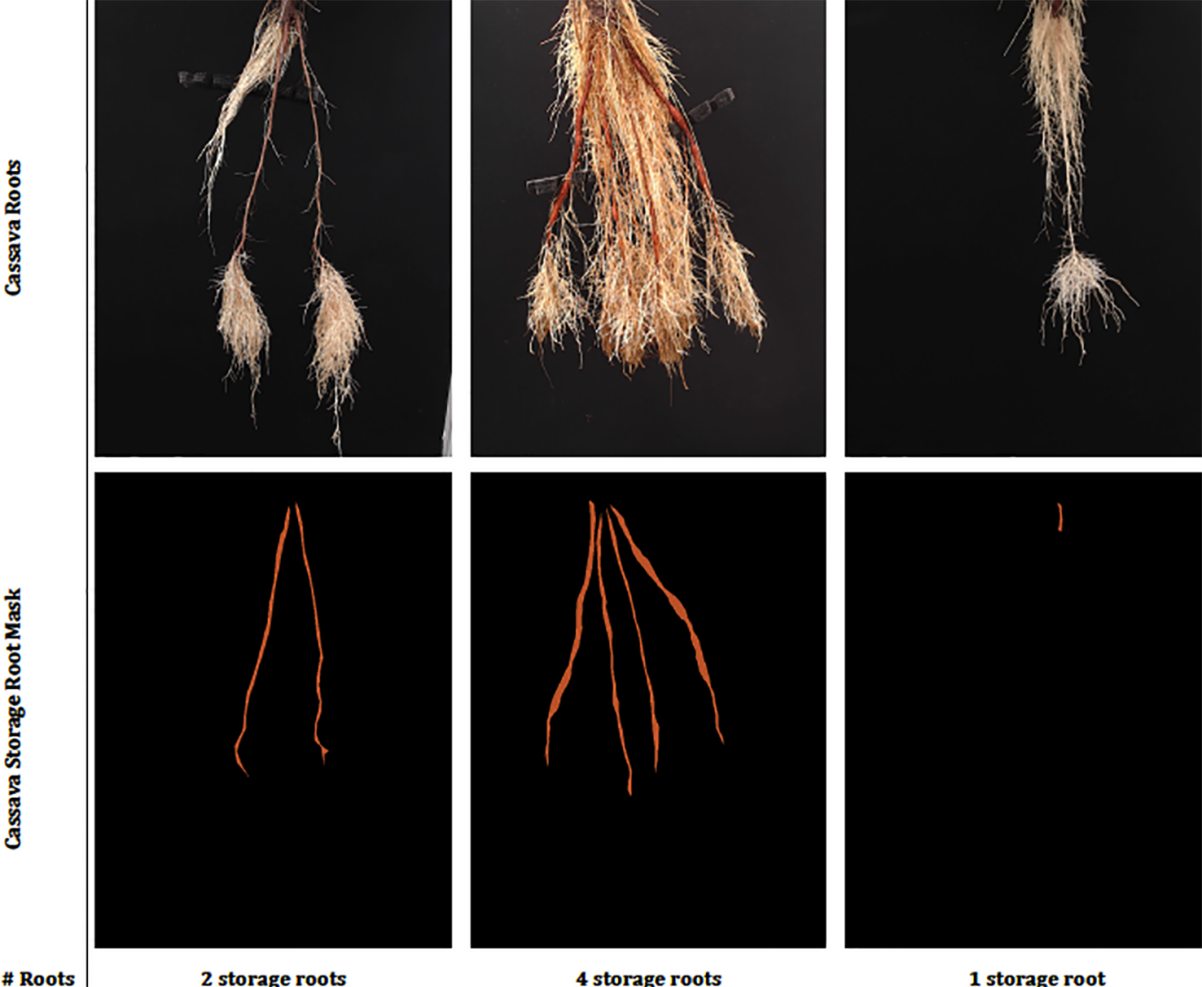
This figure shows sample real cassava root images drawn from our datasets. The top row shows the cassava root images and the bottom, the ground truth annotation (segmentation mask and number of storage roots). The challenge of identifying storage roots is particularly evident in the one storage root image.

Three semi-aeroponic systems (fog, drip and spray) designed and constructed at the International Center for Tropical Agriculture (CIAT), Colombia, were used to grow and image cassava roots (Selvaraj, 2019). Semi-aeroponic growth made it is easier to record cassava root images at regular intervals without disturbing or damaging the plants. We captured images at a resolution of 960 × 720 pixels using Logitech C922 Pro Stream Webcams with a custom-developed capture software tool, built on the OpenCV library (Intel Corporation, 2017). When imaging, the cassava plants were taken from the semi-aeroponic chambers and their roots hung carefully over a black background. Cassava experts from CIAT segmented the cassava storage roots from the captured images using a further custom-built annotation tool, and at the same time provided a manual count of the storage roots to form our annotated cassava root datasets. Each dataset was divided randomly following an "80/20" train/test split and the training data subjected to a further "80/20" train/validation split. Images were normalized by scaling their RGB values to the range 0–1. Image annotations were converted to a class label and then to a binary class matrix (one-hot encoding) before passing them to the deep neural networks. Example real data and classes can be seen in **Figure 2**.

## Image Prediction and Generation Methods

The storage root counting system proposed, and experimental evaluation conducted, here relies upon variations of two popular CNN networks: SegNet and DenseNet. In each case we reduced the number of model parameters using separable convolution, before training the resulting networks on the datasets described above, and synthetic images generated by a conditional GAN.

### Separable Convolution

MobileNet (Howard et al., 2017), MobileNetV2 (Sandler et al., 2018) and Xception (Chollet, 2017) previously used separable convolution to reduce the number of model parameters. Separable convolution reduces the number of multiplications and additions in the convolution operation, thus reducing the model's weight matrix and speeding up both the training and application of large CNNs.

A 2D convolution can be defined as in Equation 1.

(1) $$y(m,n)=\sum_{i=0}^{k-1}\sum_{j=0}^{k-1}h(i,j)x(m-i,n-j)$$

where *x* is the (*m* × *n*) matrix being convolved with a (*k* × *k*) kernel *h*. If the kernel *h* can be separated into two kernels, say *h*_1_ of dimension (*m* × 1) and *h*_2_ of dimension (1 × *n*), then the 2D convolution can be expressed in terms of two 1D convolutions as in Equation 2.

(2) $$y(m,n)=\sum_{i=0}^{k-1}h_{1}(i)\left[\sum_{j=0}^{k-1}h_{2}(j)x(m-i,n-j)\right]$$

The 2D convolution requires *k* × *k* multiplications and additions. However, separable convolution has its kernels decomposed into two 1D kernels, which then reduces the multiplications and additions to *k* + *k* and thus reduces the number of model parameters.

### Age-Prediction and CNN-Based Count Models

**Figure 3:**
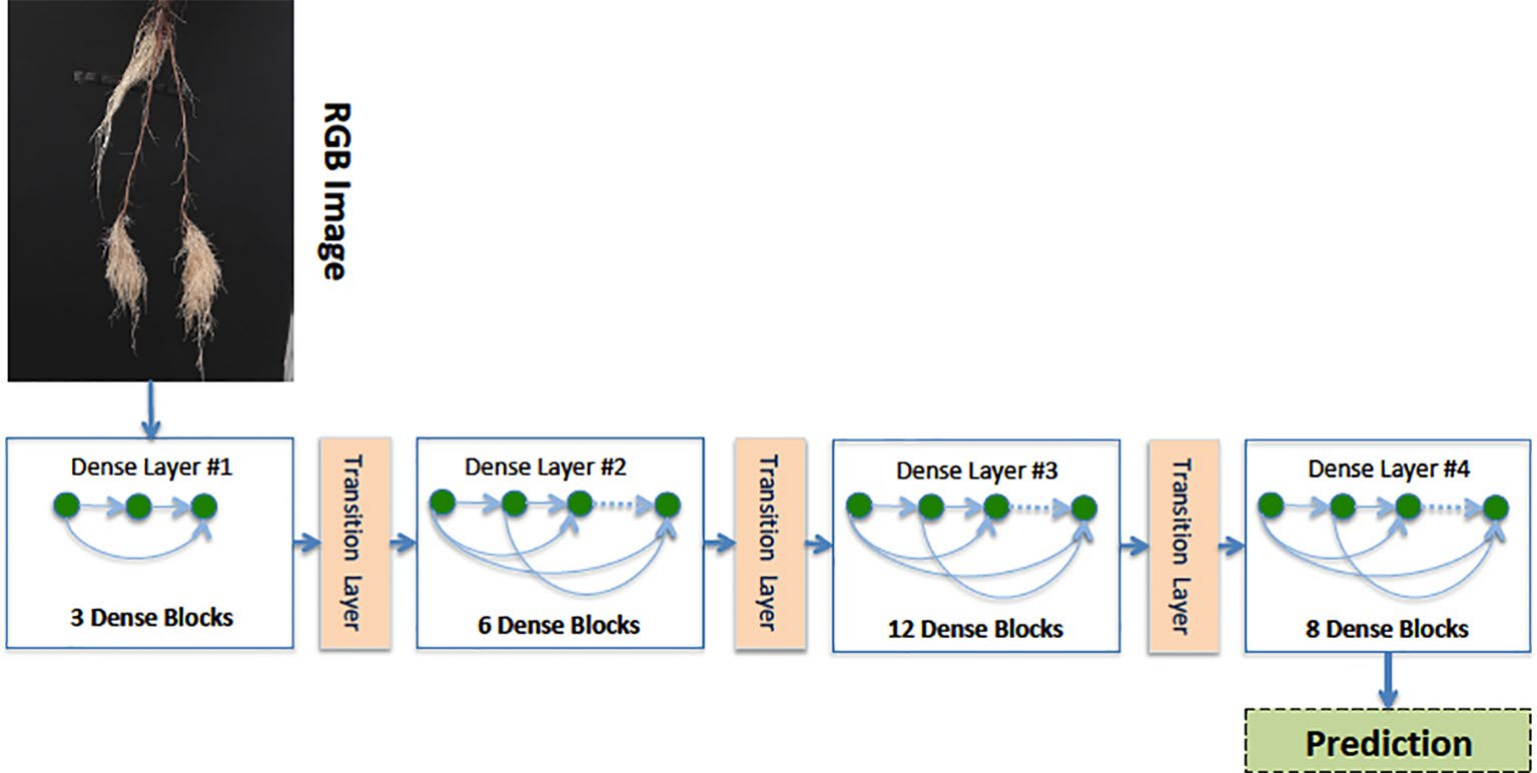
Our reduced-parameter DenseNet architecture has four dense blocks. Each dense block is made up of a 2D separable Convolution (SeparableConv2D), Batch Normalization (BN), and ReLU activations.

Our Age-Prediction and CNN-Based count models both use the DenseNet (Huang et al., 2017) architecture but with some minor changes. To decrease the model parameters, we reduced the 1 × 1 and 3 × 3 convolution blocks in the first dense block to 3, the second to 6, third to 12, and fourth to 8 and converted 2D convolutions to 2D separable convolutions. However, we used a classification layer similar to the original DenseNet: 7 × 7 global average pool, 7D fully-connected layer with a softmax activation (CNN-Based Count model only), and 2D fully-connected layer with a softmax activation (Age-Prediction model only). The reduced-parameter architecture is shown in **Figure 3**.

### Synthetic Cassava Root Generation Model

**Figure 4:**
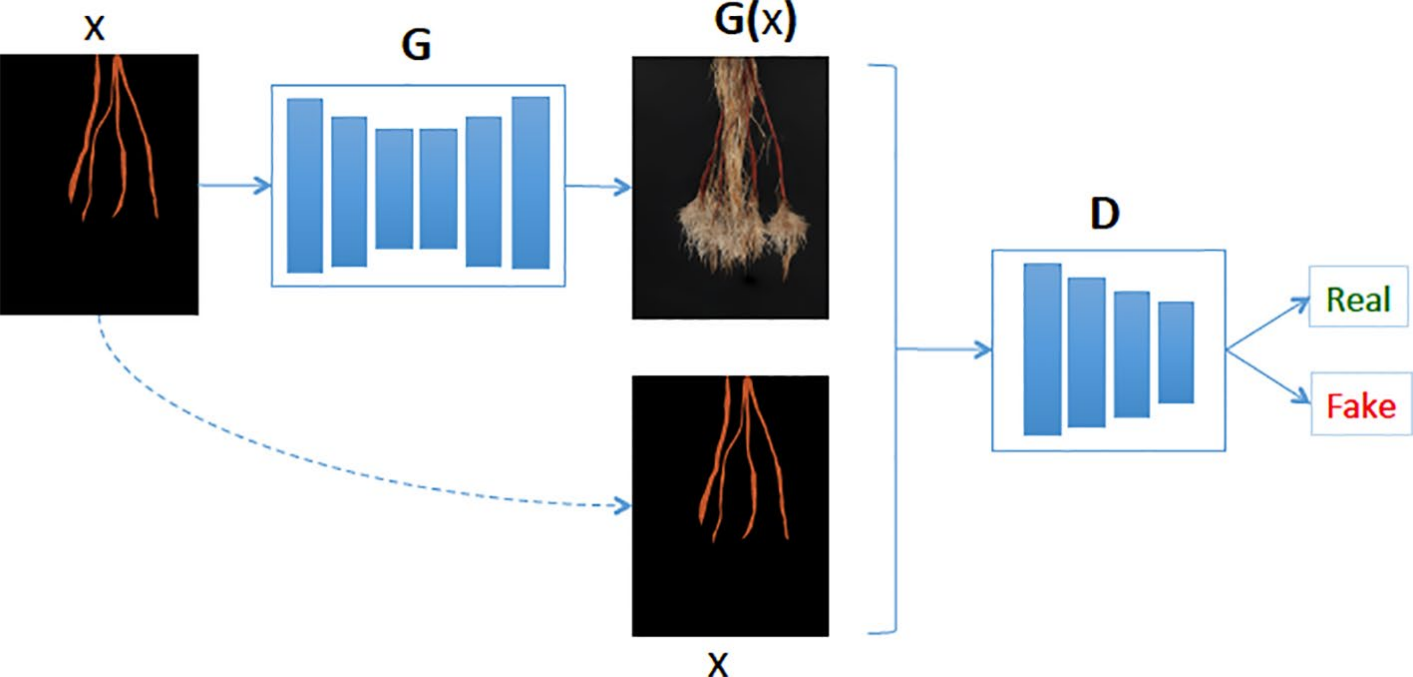
Our generative model (G) learns the mapping between cassava storage root masks and cassava roots. Then given a cassava storage root masks (x), the generator predicts the cassava root image (G(x)) and the Discriminator (D) determines if the generated cassava storage root (G(x)) is fake or real.

Our synthetic cassava root generation model uses a conditional GAN (Isola et al., 2017). The GAN comprises a generative and discriminative network chained together to make a composite model for training end-to-end. The network learns a mapping from the input mask to an output image, as well as a loss function to train the mapping (see **Figure 4**). We adapted our network architectures from those in Isola et al. (2017) and deposited the code in the GitHub repository^1^. Similar to Isola et al. (2017), our generator uses a “U-Net”-based architecture and a “PatchGAN”classifier with a patch size of 60 × 45. Both generator and discriminator use modules of the form convolution-BatchNorm-ReLu (ie. a 2D convolution followed by a Batch Normalization and then Rectified Linear Unit respectively). We use the convolution block “Conv2D-LeakyReLU-BatchNorm” (ie. a 2D convolution followed by a leaky Rectifier Unit and then a Batch Normalization respectively) denoted by *C* *_i_* and the “Convolution-Dropout-BatchNorm” block, *CD* *_i_*, where *i* is the number of filters. The convolutions are 4 × 4 spatial filters applied with stride 2. The convolutions in the encoder of both generative and discriminative models are downsampled by a factor of 2 and the decoder in the generative model up-samples by a factor of 2. Our generative model’s encoder has:

$$\begin{array}{l} C_{64}\Rightarrow C_{128}\Rightarrow C_{256}\Rightarrow C_{512}\Rightarrow C_{512}\\ \Rightarrow C_{512}\Rightarrow C_{512}\Rightarrow C_{512}\Rightarrow C_{512}\Rightarrow C_{512} \end{array}$$

The decoder has:

$$D_{512}\Rightarrow D_{512}\Rightarrow D_{512}\Rightarrow D_{512}\Rightarrow D_{512}\Rightarrow D_{512}\Rightarrow D_{256}\Rightarrow D_{128}\Rightarrow D_{64}$$

We used the BatchNorm to improve the model's training procedure, thus allowing us to use much higher learning rates. ReLU is a type of activation function, which is defined as *y = max*(0, *x*), meaning for all negative values of *x*, *y* = 0 and *y = x* otherwise. On the other hand, the Leaky ReLU has a small gradient for negative values, instead of zero (for example, *y* = 0.01*x* when *x* < 0).

The difference between our model and that in Isola et al. (2017) is that our encoder and decoder are both two blocks deeper. Here, we replace the MSE-based content loss with a loss calculated over feature maps of the VGG network, which are more robust to changes in the pixel space. We used the pre-trained VGG-16 network instead of the VGG-19. The discriminator architecture has a 60 × 45 patchGAN:

$$D_{64}\Rightarrow D_{128}\Rightarrow D_{256}\Rightarrow D_{512}$$

**Figure 5:**
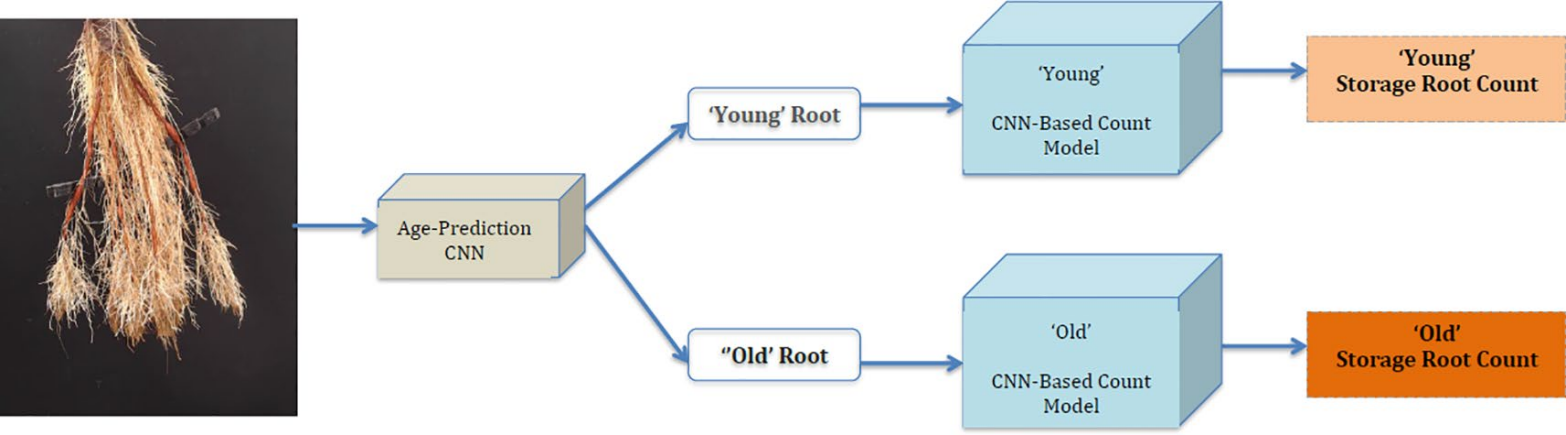
Block diagram of our Convolutional Neural Network (CNN)-Based Count Model.

Our approach, which we refer to as a "CNN-based count model," first predicts the age of the root from an image using the age-prediction model in *Age-Prediction and CNN-Based Count Models*. This prediction helps determine whether to use the "old" or "young" CNN-based model to count the number of storage roots in the given image. It should be stressed that while the architectures of these models are identical, they are trained on the distinct age datasets. **Figure 5** summarizes our method.

### Segmentation-CNN Model

**Figure 6:**
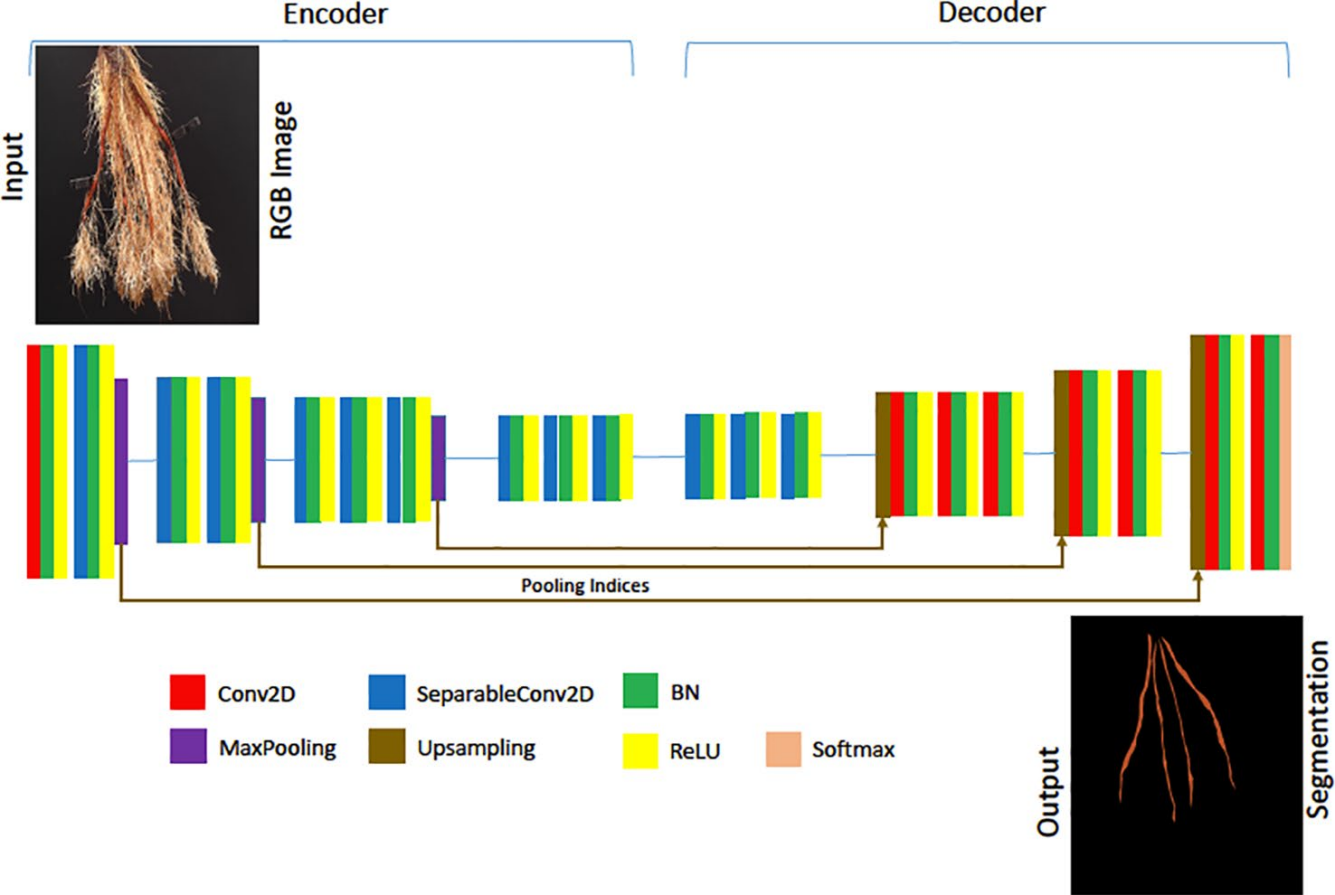
The architecture of our Lite-SegNet model is a typical VGG-16 architecture with only four blocks. The building blocks comprise of a 2D Convolution (Conv2D), 2D separable Convolution (SeparableConv2D), Batch Normalization (BN), a ReLU activation, Max Pooling, and Upsampling. This network is used to generate a reference root segmentation to help evaluate our Generative Adversarial Network (GAN) and CNN counting models.

For our deep segmentation CNN model—used in our experimental evaluation of the proposed method as a comparison approach—we used a a VGG-16 style architecture similar to SegNet (Badrinarayanan et al., 2017). We followed the convolution layers in each encoder with a batch normalization and ReLU activation except for the last block, where we placed a max-pooling at the end of each encoder block. Max-pooling is a sample-based discretization process, which down-samples the input images by reducing their dimensionality. We used the same settings as Badrinarayanan et al. (2017), with max-pooling indices for up-sampling. 2D convolutions were replaced with 2D separable convolutions to reduce the number of model parameters and produce a model similar to the Lite CNN models in (Atanbori et al., 2018). The convolution layers in both the encoder and decoder were made separable, and batch normalization and ReLU activations applied to the separated convolutions. The first convolution of the network was, however, not separated, as this captures important, high-detail features. The reduced architecture is illustrated in **Figure 6**. To provide a point of comparison with the direct prediction method, a storage root count is obtained from the segmentation mask produced by the SegNet-based CNN, following noise removal using morphological operations, by counting the number of contours in the mask.

## Experimental Evaluation

We combined the cassava root datasets detailed in *Datasets*, and the CNN architectures presented in *Image Prediction and Generation Methods* to perform the following experiments:

- The conditional GAN architecture described in *Synthetic Cassava Root Generation Model* was used to generate synthetic cassava root images from storage root segmentation masks derived from the ground truth segmentations manually created at CIAT. The similarity of synthetic and natural images was quantitatively evaluated by comparing the results of segmenting those images with the segmentation network presented in *Segmentation-CNN Model*. The hypothesis is that if synthetic and real images can be automatically segmented with similar accuracy, the synthetic data is likely suitable as additional training data when training the counting networks.
- The age-prediction network described in *Age-Prediction and CNN-Based Count Models* was used to classify real images of cassava roots as "young" or "old." The performance of this tool is evaluated in the usual way, as a classification task. The motivation for this test is to show we can identify root age with sufficient accuracy that a suitable counting model (ie. optimized for younger or older roots) can then be selected for the image in question.
- Two instantiations of the root counting network architecture described in *Age-Prediction and CNN-Based Count Models* were trained, both using real and synthetic images, to perform a direct image-to-count mapping. One of these models is trained for "young" root images, and one for "old" images. Performance of each of these networks was quantitatively compared to manually obtained ground truth. Results were also compared with identical measures of performance obtained using the segmentation-based approach described in *Segmentation-CNN Model*.

To train the image generation GAN we apply an Adam solver with a learning rate of 0.0002, and momentum parameters of *β*1 = 0.5 *β*2 = 0.999. For this experiment, we trained the network from scratch for 900 epochs and used a batch size of 2. Similar to Ledig et al. (2017), we minimize the MSE between features extracted with a pre-trained VGG19 model for real and synthetic root images. We did this because the pixel-wise loss functions such as MSE usually struggle to handle the uncertainty inherent in recovering lost high-frequency details such as texture. As input to the GAN, we supply a storage root mask, which can derive from a mask from an existing image with the same root count, or, in the case of missing data for a class (such as a lack of real data of plants with five storage roots), can be synthesized. To generate such a novel mask, masks of different root counts were combined before being passed into the GAN (see **Figure 4**).

We set the batch size of the segmentation CNN model to 2 and the age-prediction and CNN-based count models to 32. The input image resolution of the segmentation CNN is 640 × 480 pixels: large enough to maintain details of young cassava storage roots and small enough for the network to train reasonably quickly. The input image resolution of the Age-Prediction and CNN-based Count models is 256 × 256 pixels, large enough to maintain the storage root structure but again small enough for the model to train efficiently. Data augmentation applied consists of a zoom range of 0.2, brightness scaling ranging between 0.2 and 1.0, a rotation range of 10 degrees, and a horizontal flip.

We implemented all our models using Python 3.5.3 and Keras 2.0.6 with a Tensorflow backend, and trained them on a Linux server with three GeForce GTX TITAN X GPUs (12 GB memory each). Testing was carried out on a Windows 10 computer with 64GB RAM and a 3.6GHz processor.

### Metrics Used

We used the “SegNet-score,” which is similar to the "FCN-score" used in Isola et al. (2017) to quantitatively evaluate our generative model. The SegNet-scores used in our evaluation include Precision, Recall, Pixel Accuracy, and MeanIoU, previously used in Atanbori et al. (2018) to evaluate competing segmentation models. We have detailed these below:

- Pixel accuracy: This tells us about the overall effectiveness of the classifier and is defined in Equation 3.

(3) $$\frac{\sum_{i=1}^{c}n_{ii}}{\sum_{i=1}^{c}\left(\sum_{j=1}^{c}n_{ij}\right)}$$

- Mean intersection over Union (MeanIoU): This compares the similarity and diversity of the complete sample set and is defined in Equation 4:

(4) $$\frac{1}{c}*\sum_{i=1}^{c}\frac{n_{ii}}{\sum_{j=1}^{c}n_{ij}+\left(\sum_{j=1}^{c}n_{ji}\right)-n_{ii}}$$

- Average Precision: This tells us about the class agreement of the data labels with the positive labels given by the classifier and is defined in Equation 5.

(5) $$\frac{1}{c}*\sum_{i=1}^{c}\frac{n_{ii}}{\sum_{j=1}^{c}n_{ji}}$$

- Average Recall: This is the effectiveness of classifier to identify positive labels and is defined in Equation 6.

(6) $$\frac{1}{c}*\sum_{i=1}^{c}\frac{n_{ii}}{\sum_{j=1}^{c}n_{ij}}$$

where *n* *_ij_* is the number of pixels of class *i* predicted to belong to class *j*, *n* *_ji_* is the number of pixels of class *j* predicted to belong to class *i*, and *c* is the total number of classes.

We used the metrics reported in Giuffrida et al. (2015) for evaluating the leaf counting challenge to compare our CNN-based count model with the count derived from the Seg-Based Model. We choose to use these metrics since they have been widely used by the plant phenotyping community when evaluating counting models. They are:

- PercentAgreement, indicating in how many cases the algorithmic estimation agrees with ground truth
- CountDiff, average difference between algorithmic estimation of the count and ground truth, reported as mean and SD
- AbsCountDiff, average of absolute count errors, and reported as mean (SD)
- Mean Squared Error (MSE), the average squared difference between the predicted and ground truth values.

## Results

**Figure 7:**
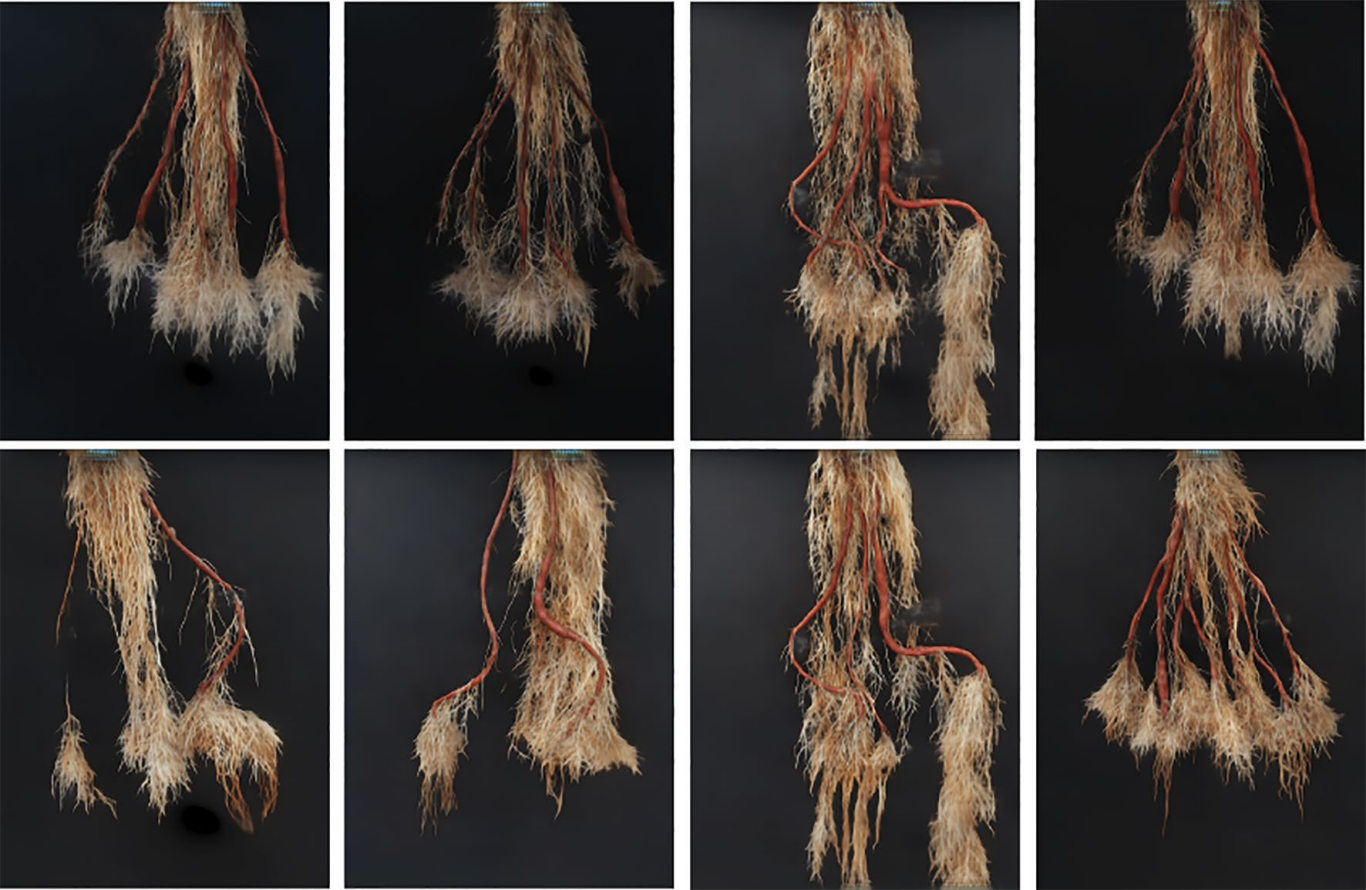
Our generative model (GAN) results: The first row show results of generated synthetic cassava roots images, all having five storage roots; no images of this class are present in the real image dataset, hence requiring synthetic generation. The last row shows results of generated synthetic cassava root images featuring a variety of storage root numbers. More examples from our GAN network can be seen in **Supplemental Images S1–S3**.

As we need to generate synthetic images to fill in classes of root numbers which are missing training images, and to augment other data-poor classes, we first examine the success of our GAN-based synthetic image generation approach. We compared our synthetically-generated cassava images (see **Figure 7** and **Supplemental Data** for examples) against real images using the SegNet-scores, which we have detailed in *Metrics Used*, and present the results in **Table 1**. The SegNet-scores we considered are Precision, Recall, Pixel Accuracy, and MeanIoU.

**Table 1:** Comparison of segmenting synthetically generated cassava images versus the real images using the SegNet-scores: Precision, Recall, Pixel Accuracy, and MeanIoU.

Data	Precision	Recall	Pixel accuracy	MeanIOU
Real	99.30%	99.30%	99.30%	70.35%
Synthetic	98.38%	98.36%	98.48%	65.56%

The results reported were based on using the segmentation model trained on real images, for testing real and synthetically generated cassava images. The reasoning is that if the synthetic images are sufficiently similar to real images, then the model should be able to segment the synthetic images with comparable accuracy; if the generated images are visually different to the real images, segmentation will fail. From **Table 1**, it can be seen that although the SegNet-scores of the synthetically generated cassava images are lower than those associated with real images, the difference is small—only 4% based on the MeanIoU, and even smaller when considering the other metrics. From this, we observe that if the synthetic images can be segmented almost as well as the real images, they will be suitable as synthetic data to replace missing real images in the training data for the counting CNN procedure.

In order to choose an appropriate counting CNN model, we must estimate the age of the plant in an image as either young or old. Therefore, we next evaluate classifying cassava images from each dataset into featuring either old (≥ 2.5 months) or young (< 2.5 months) cassava roots using our age-prediction model. Results are presented in **Table 2**. These results are then used as input into our CNN-based count model to count storage roots. Storage roots of an image predicted as "young" are counted using the CNN-based model trained on "young" cassava roots whereas those predicted as "old" are counted by the model trained on old cassava roots.

**Table 2:** Percentage of correctly classified images for young and old root classes, using the age-prediction model.

	Test-split	Test data	Combined
Old	95%	71%	83%
Young	99%	100%	100%
**Overall**	**97%**	**86%**	**91%**

Test-split represents data from the train/test split, test data is taken from the field after building the models, and combined data comprises both test-split and test data.

**Figure 9:**
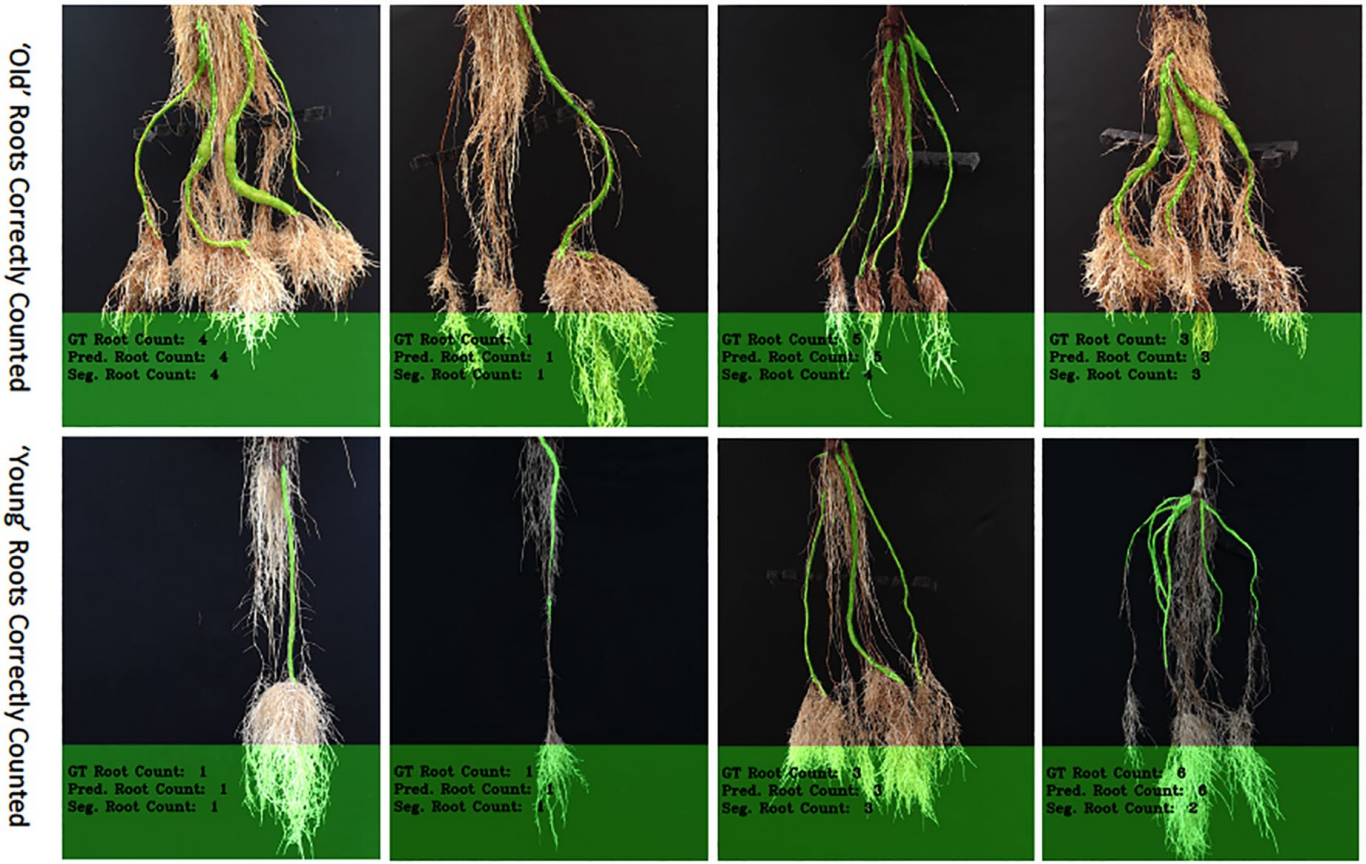
The figure shows correctly counted storage roots, with the top row showing "old" storage roots and the bottom, "young" ones. More examples of outputs for the counting networks can be seen in **Supplemental Images S10–S15**.

We then evaluated our CNN-based cassava storage roots counting model, comparing counts generated by our CNN model with counts generated from an image processing pipeline deriving from the Seg-Based model. We evaluated using the test-split (data from train/test split), test data (taken from the field after building the models), and combined test data (both test-split and test data). The results are presented in **Tables 3**, **4**, and **5**, respectively, and some good quality example outputs are shown in **Figure 9** (error cases are raised in the discussion).

**Table 3:** Comparison of storage root-counting accuracy for our proposed Convolutional Neural Network (CNN)-based approach versus a more traditional segmentation-based approach.

		% Agreement	CountDiff	ABS​CountDiff	MSE
CNN-Based	Old	90%	0.17 ± 0.79	0.26 ± 0.76	0.65
	Young	74%	0.08 ± 0.84	0.38 ± 0.76	0.72
Seg-Based	Old	54%	1.39 ± 1.92	1.39 ± 1.92	5.61
	Young	49%	1.04 ± 1.54	1.08 ± 1.52	3.45

Results from the Test-split data; this is the data from train/test split that was reserved for testing the model. We report: %Agreement: the higher the better, CountDiff and ABSCountDiff as mean ± SD: the smaller the better and MSE: the smaller the better.

**Table 4:** Comparison of storage root-counting accuracy for our proposed CNN-based approach versus a more traditional segmentation-based approach.

		%​Agreement	CountDiff	ABS​CountDiff	MSE
CNN-Based (Ours)	Old	70%	0.27 ± 0.96	0.45 ± 0.89	1.00
	Young	62%	0.36 ± 1.59	0.79 ± 1.25	3.18
Seg-Based	Old	20%	0.09 ± 1.31	1.00 ± 0.85	1.72
	Young	46%	1.14 ± 1.25	1.14 ± 1.25	3.64

Results here are from new isolated test data after the model was built. We report: percent agreement: the higher the better, CountDiff and ABSCountDiff as mean ± SD: the smaller the better and MSE: the smaller the better.

**Table 5:** The table shows results from combining the Test and test-split Data.

		%​Agreement	CountDiff	ABS​CountDiff	MSE
CNN-Based (Ours)	Old	86%	0.12 ± 0.77	0.24 ± 0.74	0.61
	Young	71%	0.14 ± 1.06	0.47 ± 0.96	1.14
Seg-Based	Old	47%	0.84 ± 1.63	1.04 ± 1.51	3.37
	Young	48%	1.08 ± 1.49	1.11 ± 1.47	3.38

We reported %Agreement—the higher the better, CountDiff and ABSCountDiff as mean ± SD—the smaller the better and MSE—the smaller the better.

The CNN-based model (ours) outperformed the Seg-Based system based on all metrics and test data splits reported. The difference is more than 36% for "old" roots and 25% for "young" roots based on the test-split data only. However, based on the combined test data (both test and test-splits), this is 39% and 23%. In all cases our CNN-based approach outperforms the segmentation-based approach. We present a discussion of these results in *Discussion*.

### Discussion

We generated synthetic images for only the "old" cassava storage roots (≥2.5 months). We did this since our model requires all classes of the dataset (ie. all possible numbers of storage roots) to be present, but this age category had some missing. Based on the "SegNet-Scores," our synthetically generated cassava images are comparable to real images and therefore should be able to be used to train our CNN-based count models. **Figure 7** shows the results of synthetic images generated using our generative model. Visually inspecting these images also show that they are comparable to the real images (c.f. **Figure 1**). Even though the synthetically generated images were high resolution (960 × 720), they were less blurry, since our perceptual loss function, which uses the high-level feature maps of the VGG network adopted from Ledig et al. (2017) has been shown to produce less blurry images than an L1-Loss function, for example. We thus effectively use the generated images to supplement the real ones for training our count-model.

**Figure 8:**
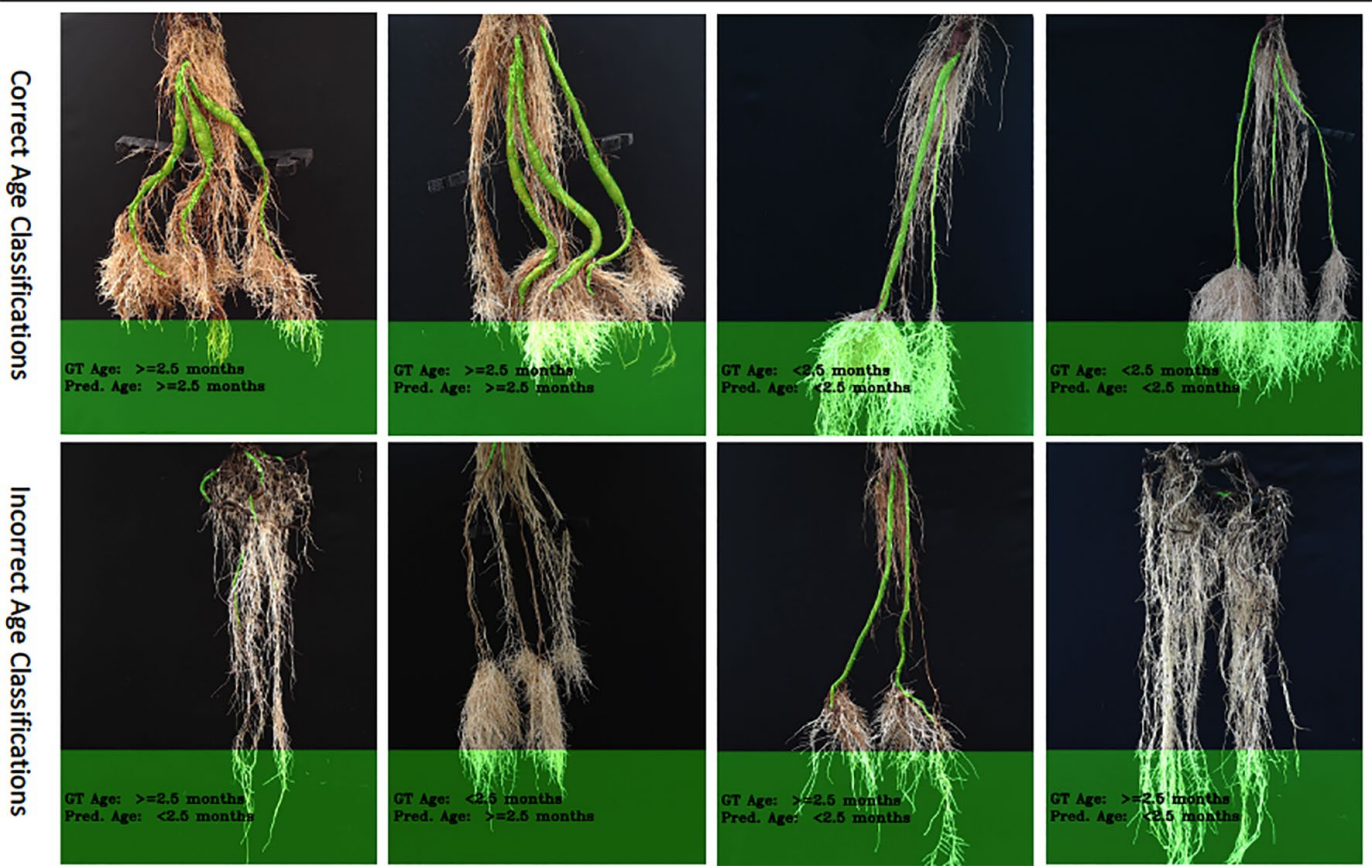
Example results of our age-prediction model. Top row shows images of correctly predicted age; bottom row shows incorrect predictions. More age prediction example images can be seen in **Supplemental Images S4–S9**.

The correct prediction rate of our age-prediction model on our "old" roots is 83%, which by itself can be considered a good result. However, we observed that the model performs better in correctly predicting "young" cassava roots than the "old" ones. This is evidenced in the correct prediction rates reported in **Table 2**. The difference in the correct prediction rate between "young" and "old" roots is 4% on the test-split data, 29% on the test data collected after training the model, and 17% based on the combined test sets. We have also shown results of some correctly and incorrectly predicted ages of cassava storage root in **Figure 8**. Most of the incorrect predictions lie closer to the boundary age (2.5 months) of "old" and "young" roots. The second and third images in row two of **Figure 8** shows some of these types of incorrect age predictions. Other incorrectly classified images were varieties of cassava roots that were poorly represented in the training data (images 1 and 4 in row two of **Figure 8**).

The "old" cassava roots are usually correctly counted by the CNN-based model (ours) and Seg-Based model. "Young" roots with well-defined storage roots are also very likely to be correctly counted by both models. **Figure 9** shows the results of correctly counted storage roots. The top row shows images of the "old" cassava roots that are correctly counted and the bottom the "young" ones. Clearly, "young" roots with well-defined storage roots are correctly counted by both models [especially the CNN-Based model (ours)]. However, overall, counting the "old" cassava storage roots are more successful than the "young" ones as their storage roots are well-defined.

**Figure 10:**
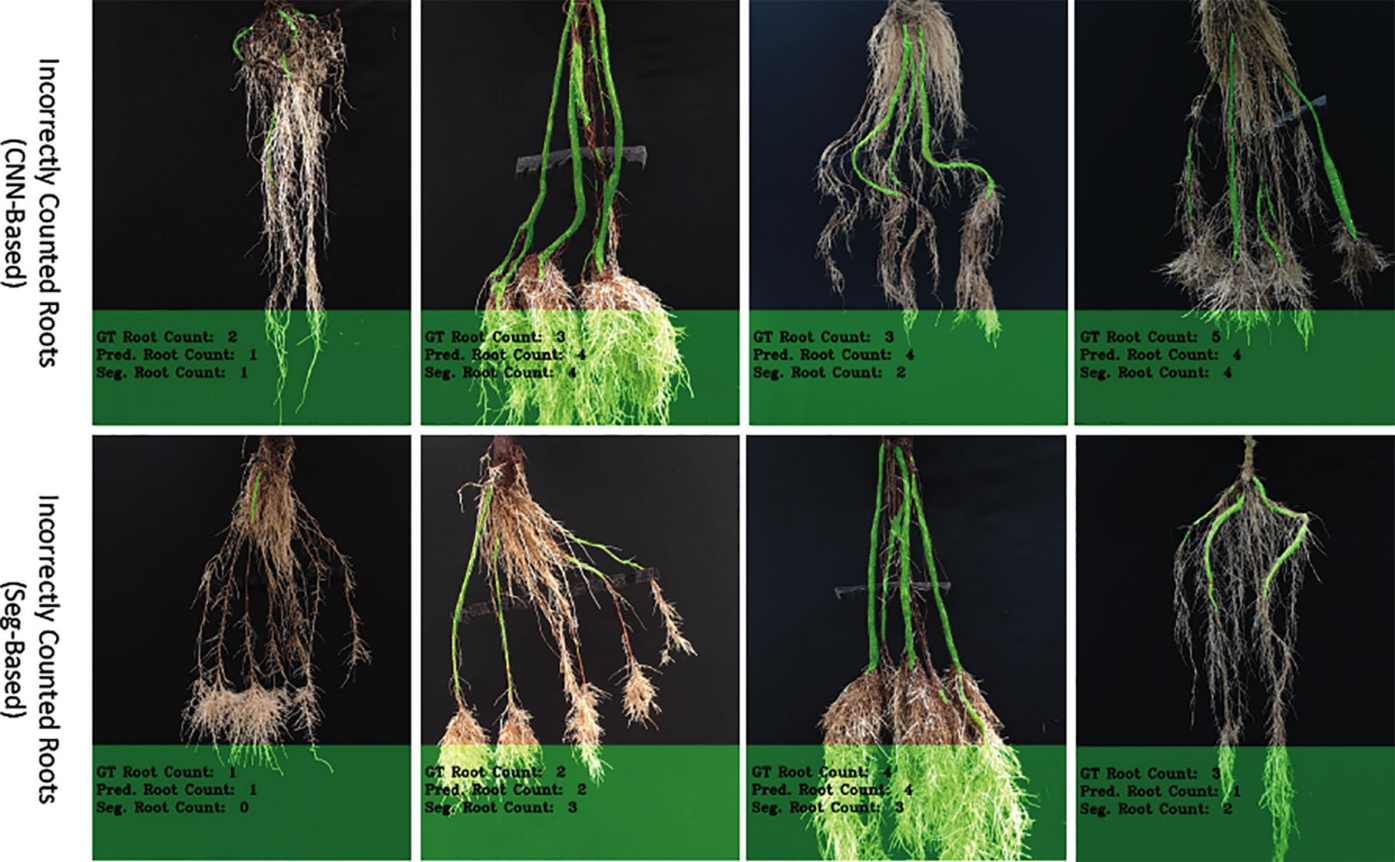
The figure shows incorrectly counted storage roots, with the top row showing results from the CNN-based model and the bottom, the Seg-based model.

We have observed that our CNN-based counting model outperforms the Seg-based model substantially based on both datasets. The reason for this difference is that the Seg-based model uses the masks of cassava storage roots, which sometimes overlaps, thus making them difficult to count. However, because the CNN-Based model (ours) does not rely on segmented masks, it is usually more successful on this type of images. Furthermore, the "Seg-Based" model fails to correctly count the storage roots when there is incorrect segmentation from the segmentation-CNN model. There are also additional cases when both our "CNN-Based" and "Seg-Based" models incorrectly count roots (see **Figure 10**). Again, this happens more in "young" roots where storage roots are visibly harder to pick out, and also varieties of cassava that are under-represented in the training set. Perhaps more insight could be gained into these errors by building in an explainable approach to the deep learning. To elucidate why decisions are made by the deep learning system, future systems will attempt to reveal to the user regions which are used in the counting process. Understanding the exact mechanism of GAN-based image generation is more challenging, and is a focus of current research.

## Conclusion

We proposed two convolutional network architectures for counting "old" and "young" cassava storage roots, which we refer to as "old" and "young" CNN-Based count models respectively. Since we needed two models, we further proposed a CNN-based age-prediction architecture that first classifies storage roots as either "old" or "young" and then use the appropriate CNN-based count model to predict the number of storage roots. In our experiments, the age-prediction model achieved a state-of-the-art prediction accuracy on both datasets. We evaluated our CNN-based count model with a similar approach that uses a segmentation based method, and it outperformed it considerably.

We generated synthetic images for missing count classes in the "old" root dataset since our approach requires data for all classes, and found that they are comparable (both visually and when automatically segmented) to the real mages. We investigated incorrect counting by our model and found they were often caused by storage roots lying closer to the boundary age (2.5 months) used to separate "old" and "young" roots. We also found some incorrect classifications caused by testing varieties of cassava roots that were few or missing from the training data. As future work, we propose to collect more data for each variety of cassava roots in our dataset, which will help improve our models' performance. Even though we can generate these images with our conditional GAN, this also requires more data to produce realistic images. We also propose to design additional CNN-Based models that will predict the total length and volume of cassava storage roots, which will help us develop a complete image-based cassava root phenotyping system. The approach here has been developed to support cassava phenotyping work, in particular to support the development of a low-cost aeroponic phenotyping system. Future work will need to consider the ease of transfer from this system to other, similar systems, and a transfer learning approach may be required to update models for new image sets.

## Data Availability Statement

The raw data supporting the conclusions of this manuscript will be made available by the authors, without undue reservation, to any qualified researcher.

## Author Contributions

JA was primary author and developer of the models and experiments. AF and TP supervised JA, and assisted with model development and manuscript writing. MM and MS provided biological expertise and data collection and annotation in Colombia.

## Funding

This work was supported by the Biotechnology and Biological Sciences Research Council [BB/P022790/1].

## Conflict of Interest

The authors declare that the research was conducted in the absence of any commercial or financial relationships that could be construed as a potential conflict of interest.

## Footnotes

1 https://github.com/Amotica/CNN-Based-Cassava-Storage-Roots-Counting/tree/master/rootGAN

## Supplementary Material

**Supplementary Figure 1–15** are available. These detail more example images of GAN generation, age prediction, and storage root counting.
