# Supplementary material for: Convolutional Neural Net-Based Cassava Storage Root Counting Using Real and Synthetic Images
Source: Front Plant Sci. 2019 Nov 26;10:1516. doi: 10.3389/fpls.2019.01516 (PMC6888701; doi:10.3389/fpls.2019.01516)
Supplement: Supplementary file 1 [file DataSheet_1.pdf]

Supplementary Material

1 SUPPLEMENTARY FIGURES

This supplementary material contains additional figures of our experimental results from our synthetic image generation model, age-prediction model and CNN-based count models.

1.1 Figures of Synthetically Generated Cassava Storage Roots.

We generated synthetic images for only the ”old” cassava storage roots ($\geq$ 2.5 months). We did this since our counting model requires all classes of the dataset to be present, but some classes of counts in the ”old” dataset had some missing. We have presented additional figures of GAN generated results in Figures S1 - S3.

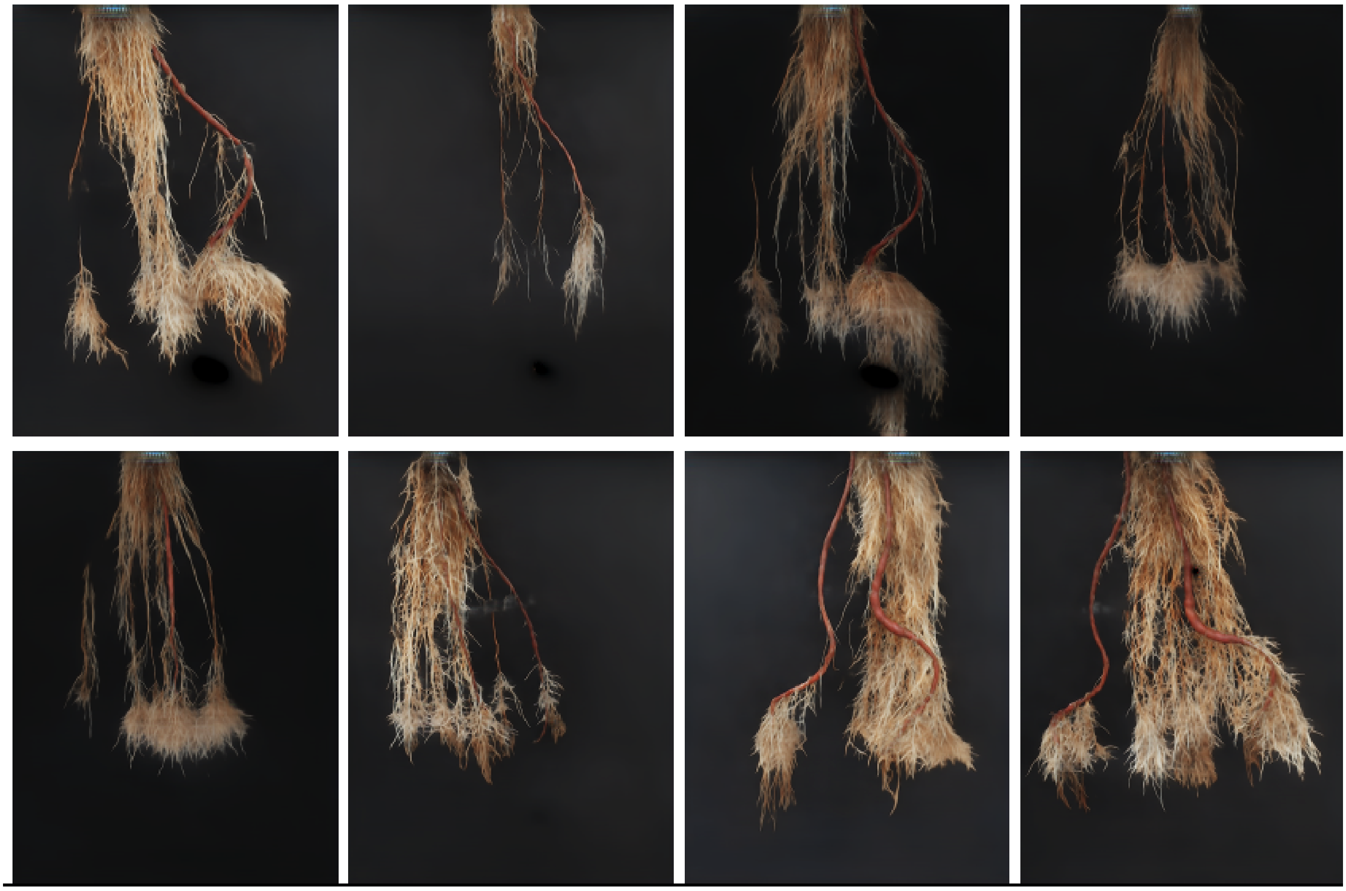

Figure S1: Conditional GAN results.

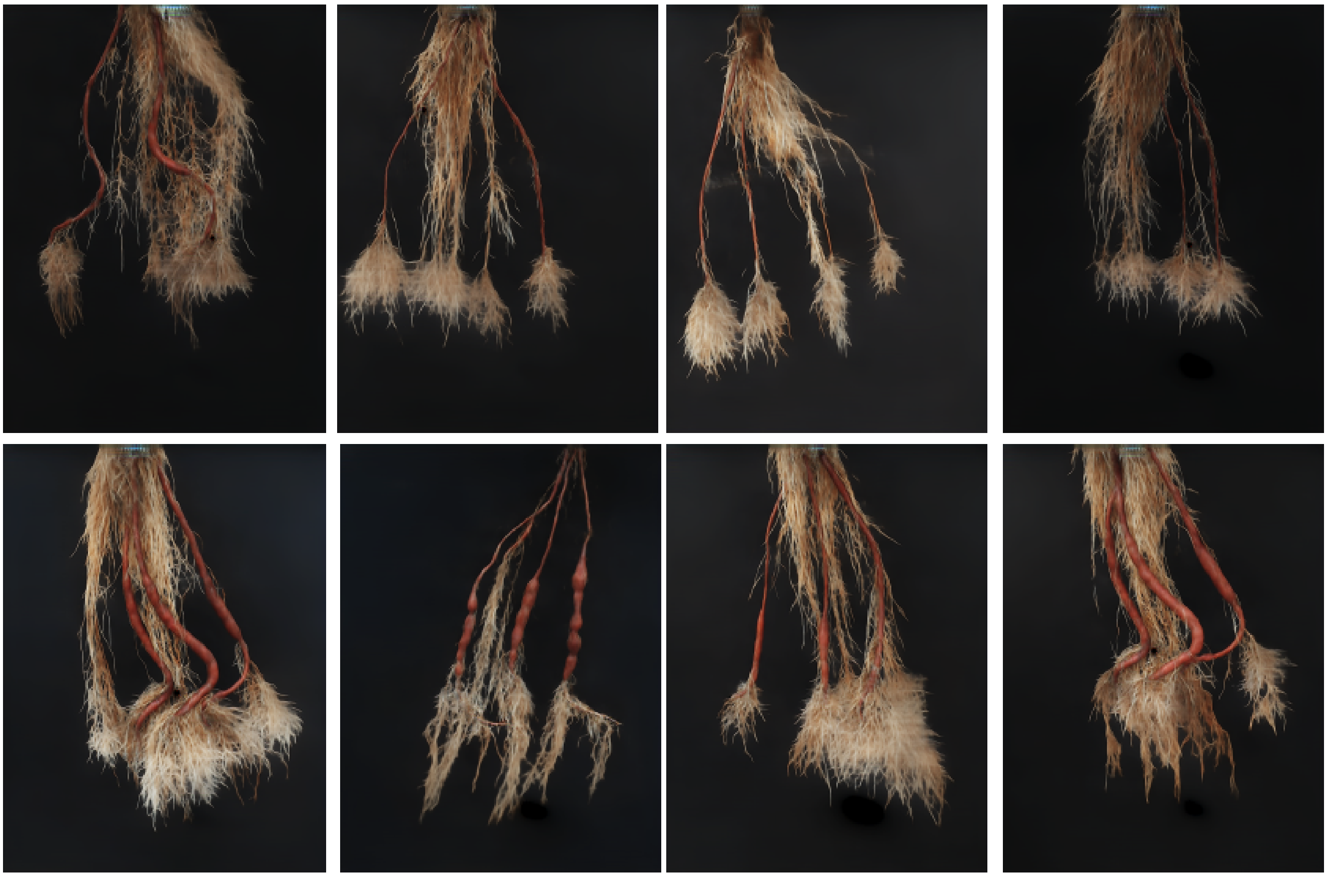

Figure S2: Conditional GAN results.

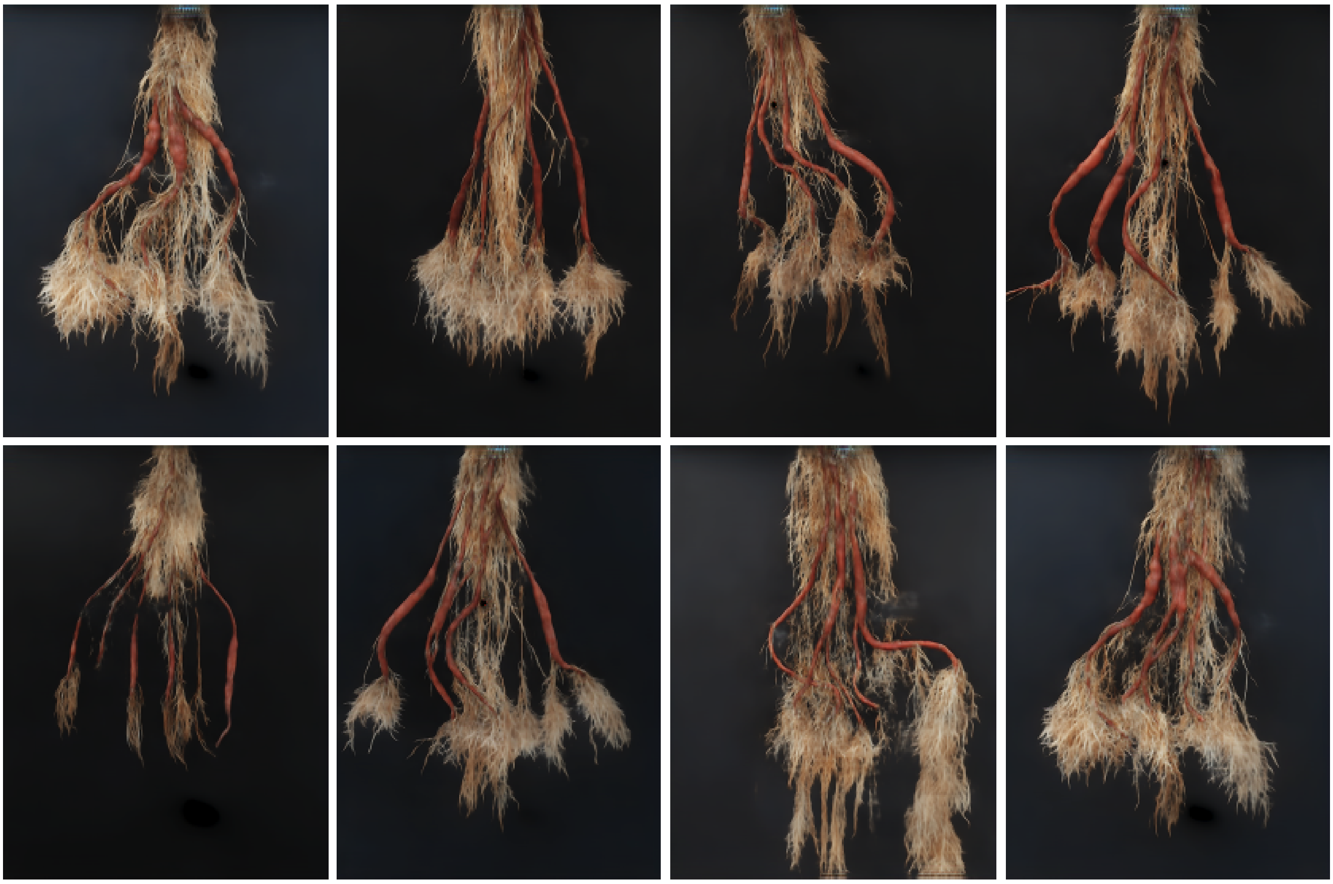

Figure S3: Conditional GAN results.

1.2 Figures of Age-Prediction of Cassava Roots.

We classify cassava images from both datasets into either old ($\geq 2.5months$) or young ($< 2.5months$) cassava roots using our age-prediction model and present the additional result as supplementary figures in Figures S4 - S9. Figures S4 - S6 represents prediction results where the ground truth is ”old” cassava roots ($\geq 2.5months$) and Figures S7 - S9 prediction results where the ground truth is ”young” cassava roots ($< 2.5months$).

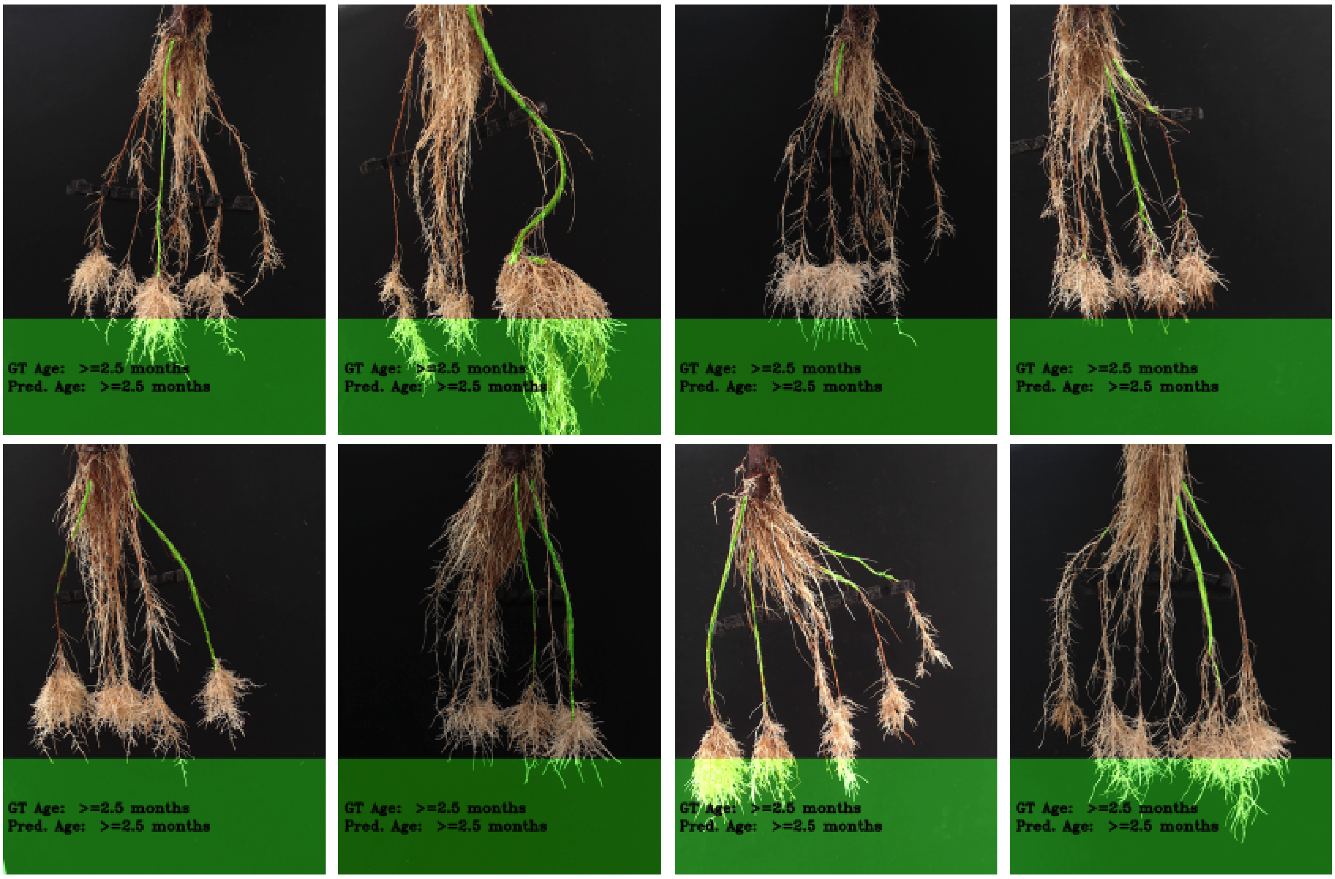

Figure S4: The figure shows a first set of supplementary results of our age-prediction model based on the **”old” cassava root dataset**.

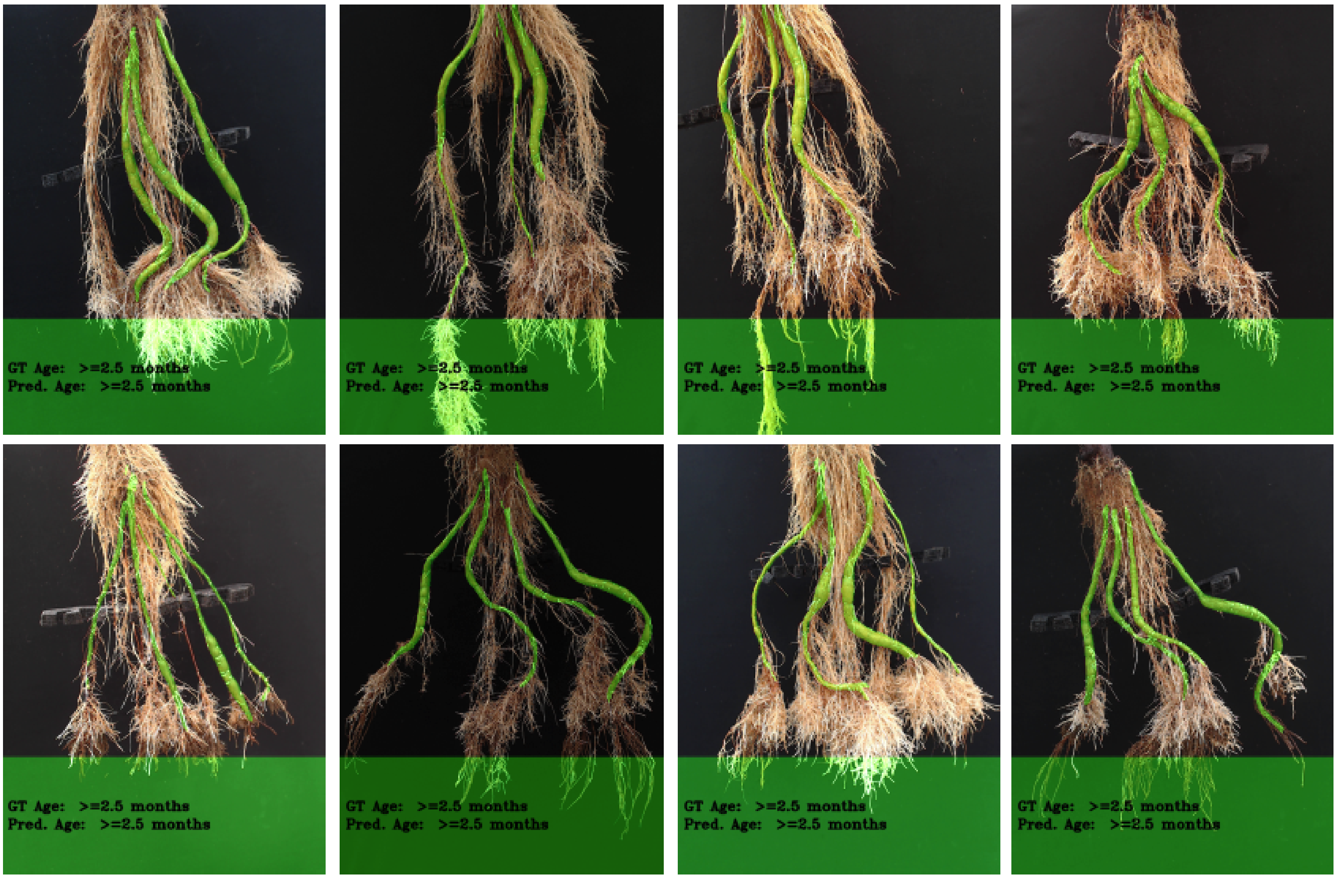

Figure S5: The figure shows a second set of supplementary results of our age-prediction model based on the **"old" cassava root dataset**.

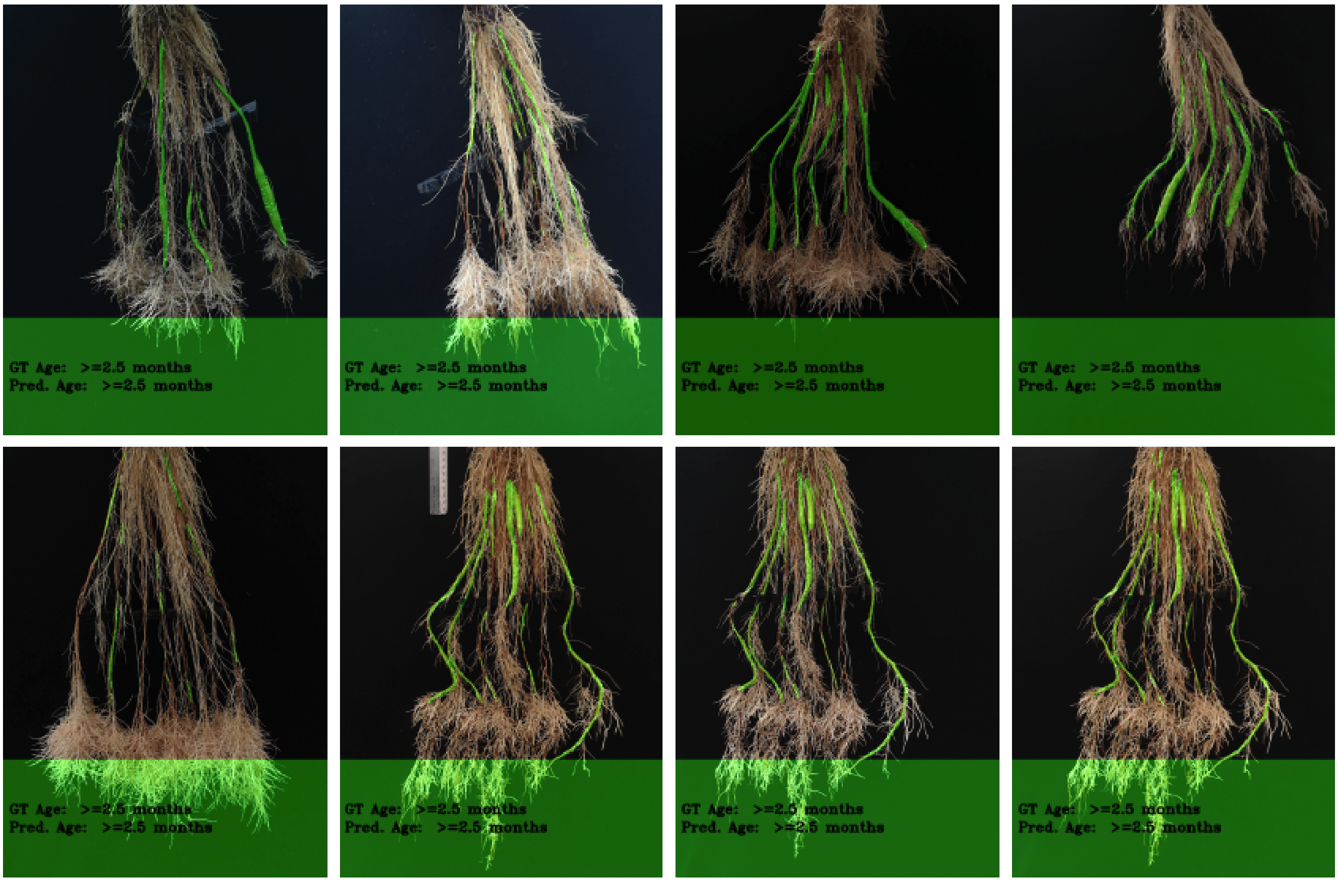

Figure S6: The figure shows a third set of supplementary results of our age-prediction model based on the **”old” cassava root dataset**.

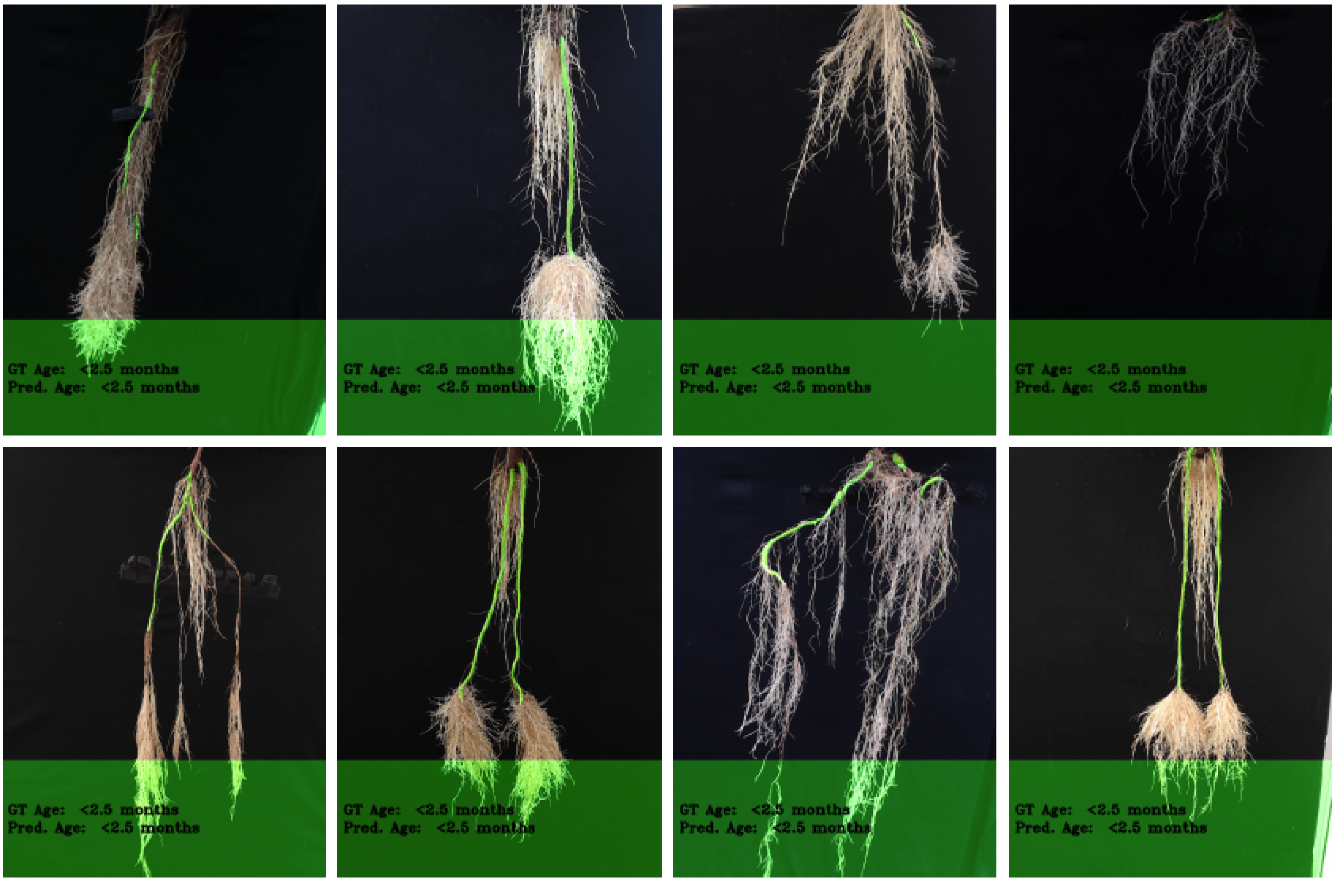

Figure S7: The figure shows a first set of supplementary results of our age-prediction model based on the **"young" cassava root dataset**.

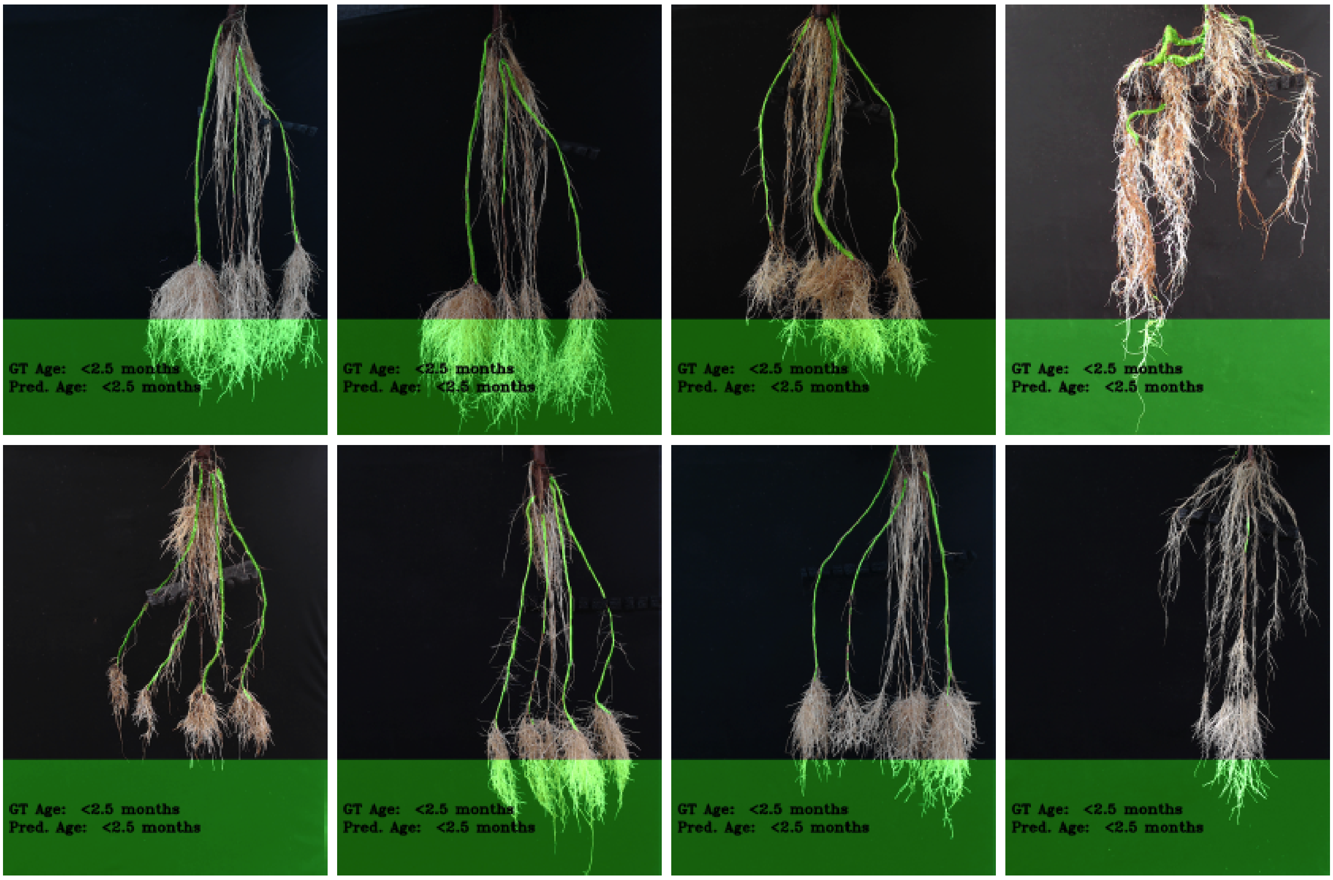

Figure S8: The figure shows a second set of supplementary results of our age-prediction model based on the **”young” cassava root dataset**.

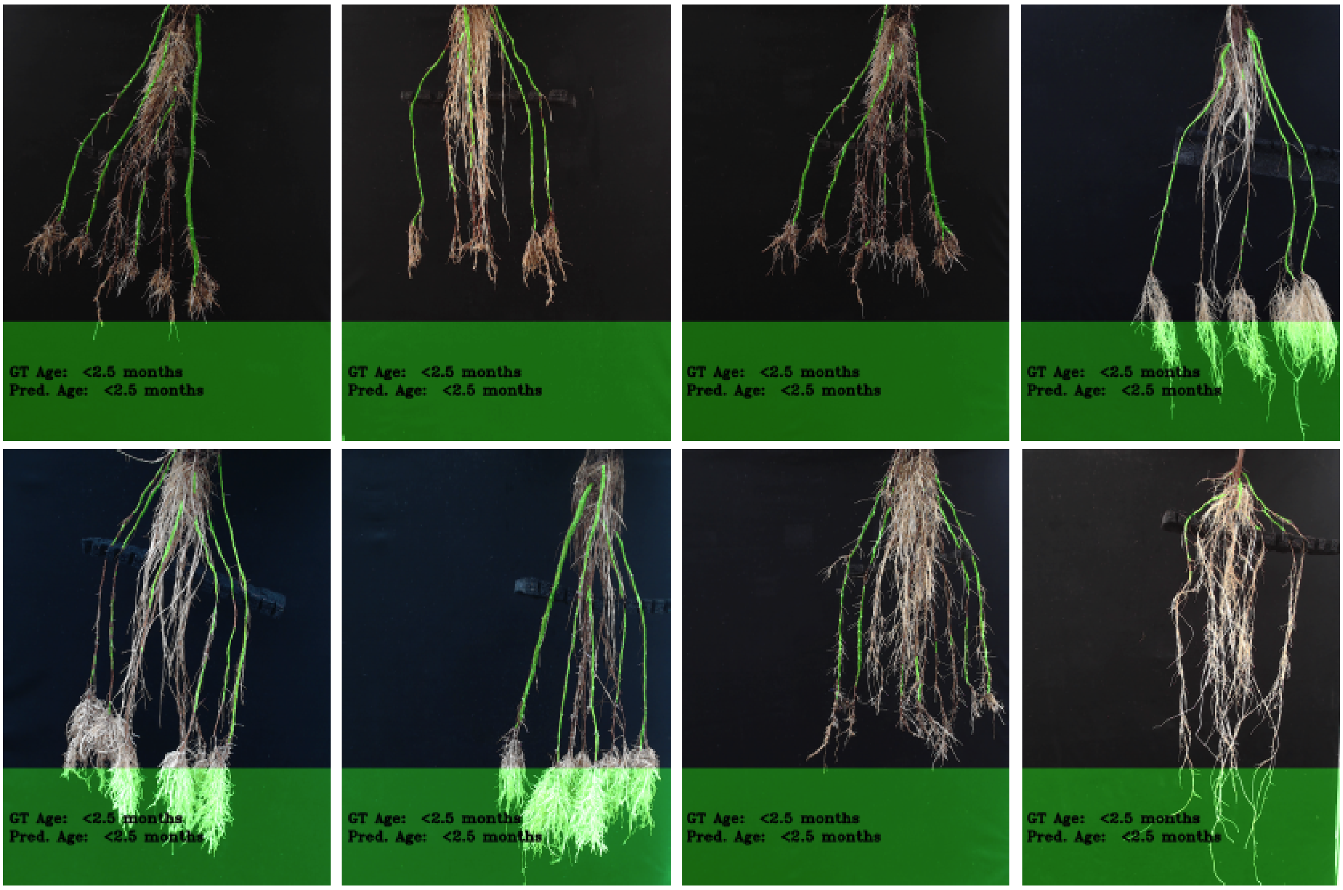

Figure S9: The figure shows a third set of supplementary results of our age-prediction model based on the **”young” cassava root dataset**.

1.3 Figures of CNN-based count of Cassava Roots.

Figures S10 - S15

We count storage roots using our CNN-Based cassava storage roots counting model and the Seg-Based model using the test-split (data from train/test split), test data (taken from the field after building the models), and combined test data (both test-split and test data). We provide additional data in S10 - S15. Figures S10 - S12 represents count results where the ground truth is ”old” cassava roots ($\geq 2.5 months$) and Figures S13 - S15 count results where the ground truth is ”young” cassava roots ($< 2.5 months$).

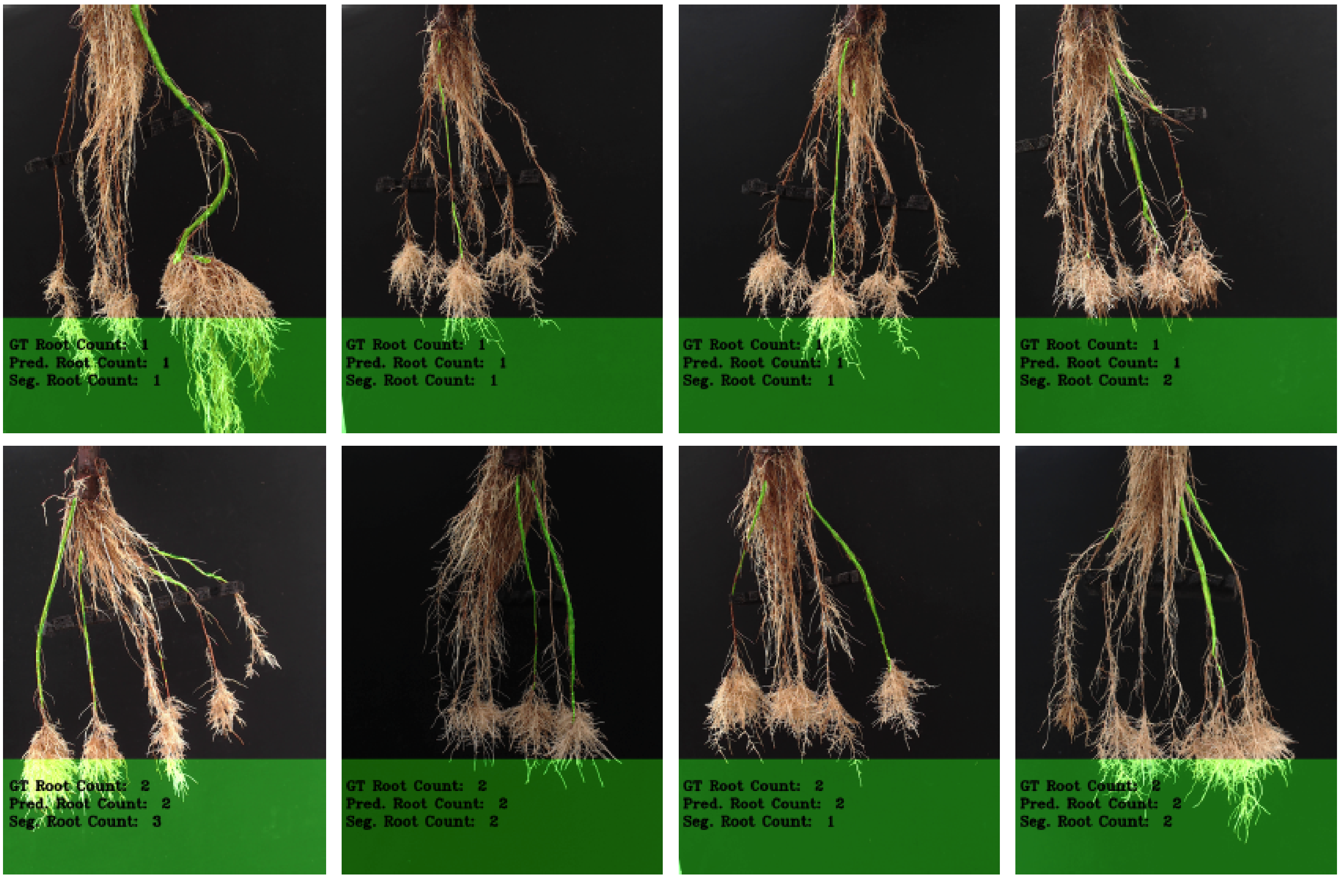

Figure S10: The figure shows a first set of supplementary results of counted storage roots from our CNN-based using our **”old” cassava root dataset**.

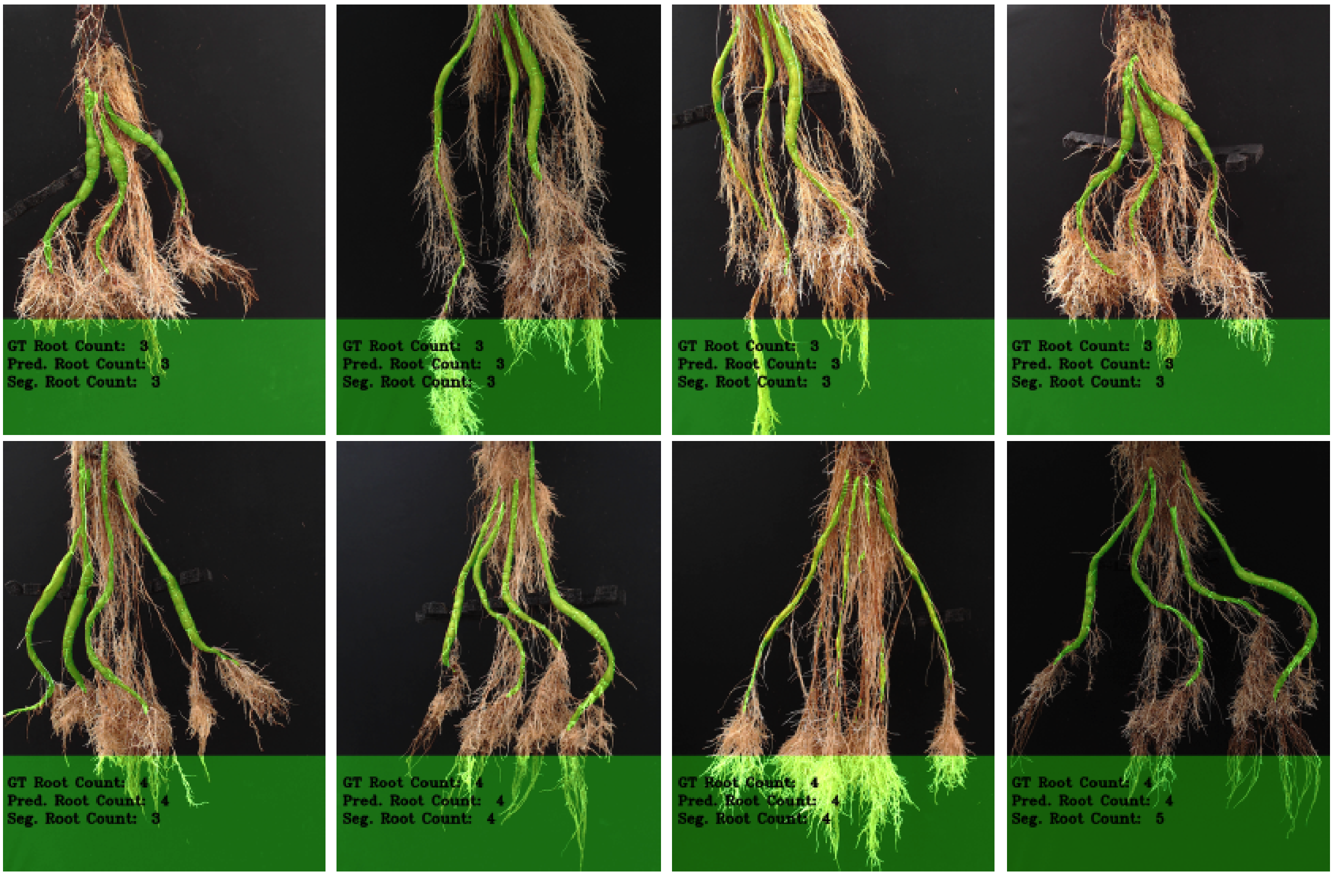

Figure S11: The figure shows a second set of supplementary results of counted storage roots from our CNN-based using our **”old” cassava root dataset**.

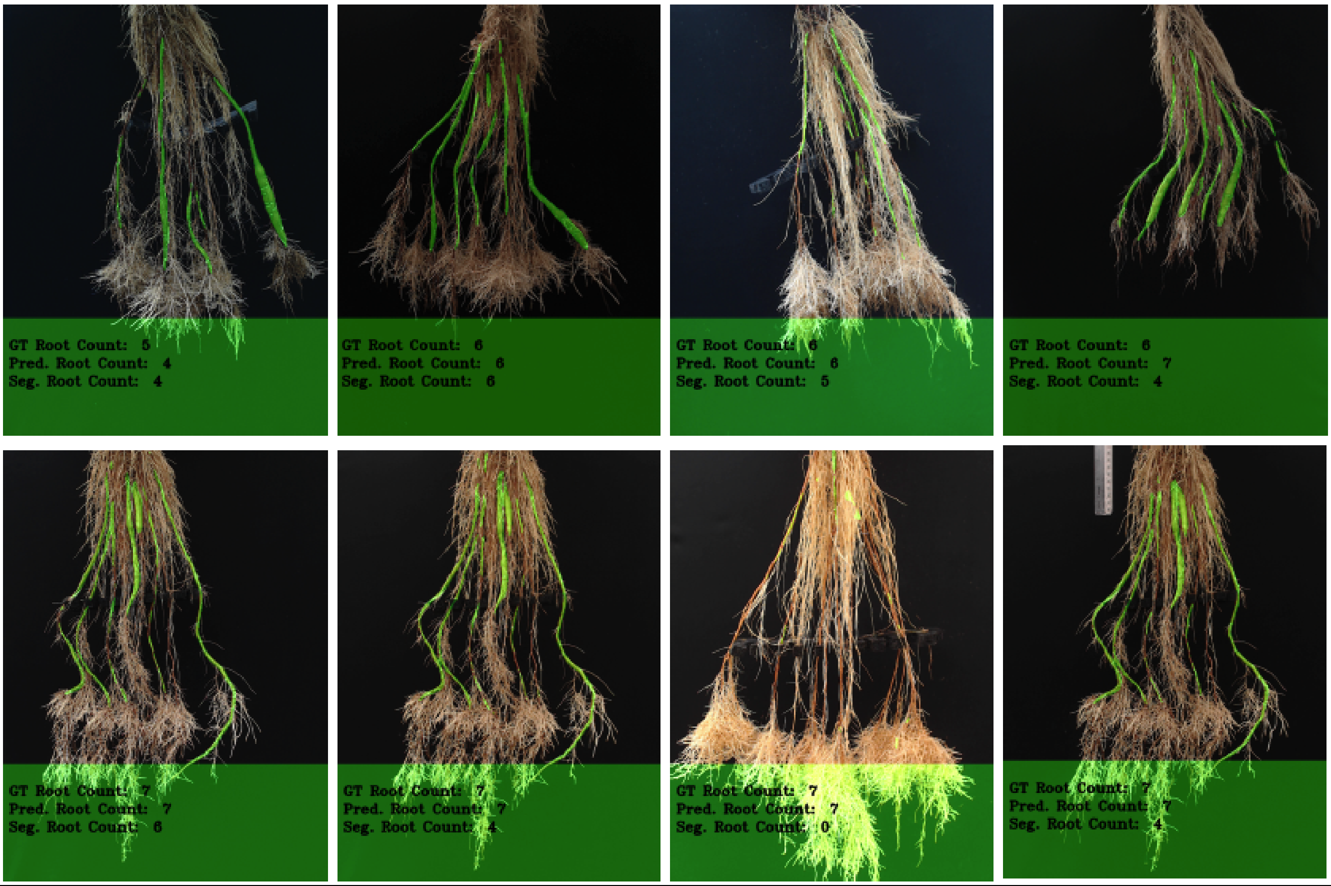

Figure S12: The figure shows a third set of supplementary results of counted storage roots from our CNN-based using our **”old” cassava root dataset**.

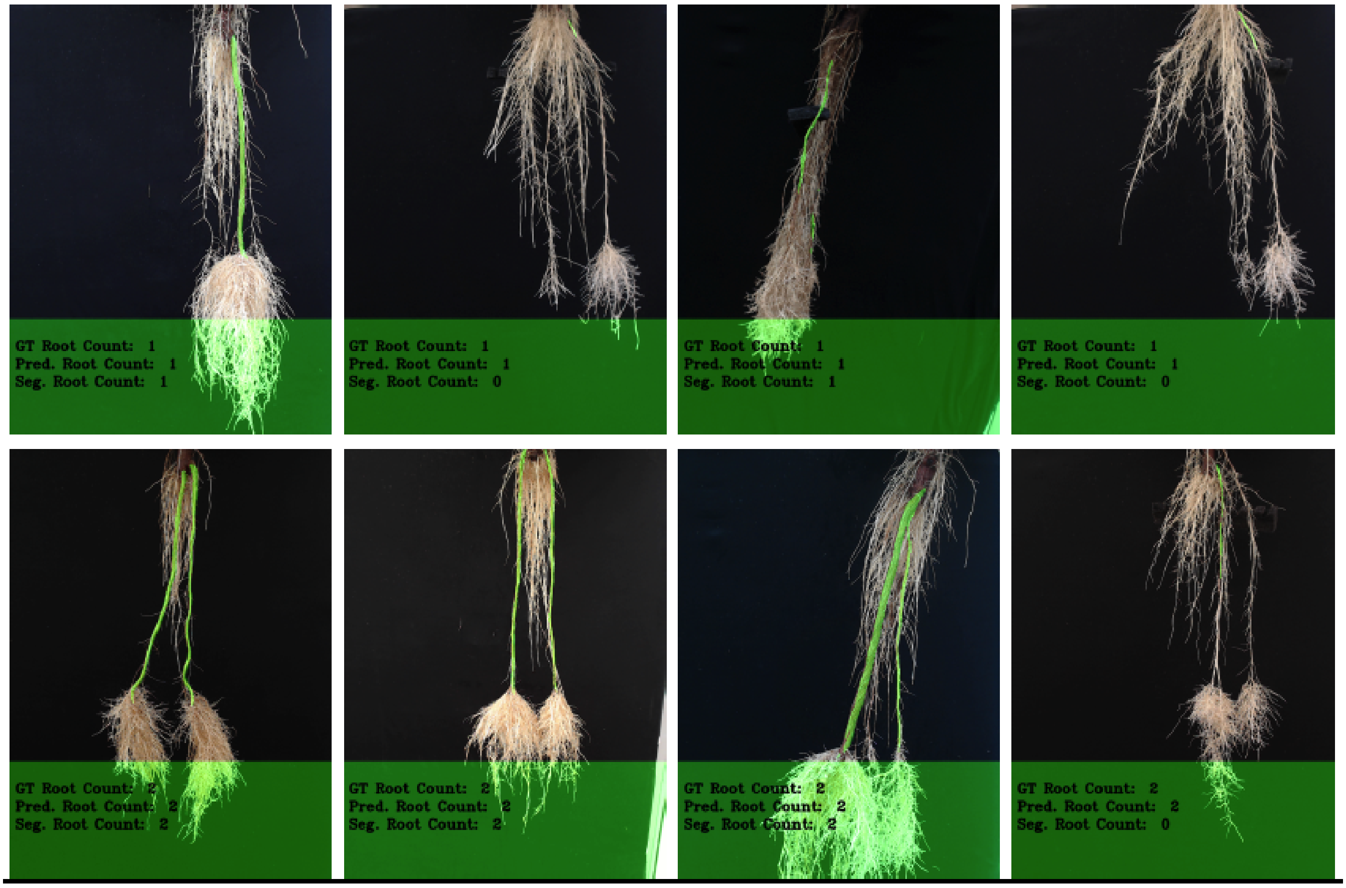

Figure S13: The figure shows a first set of supplementary results of counted storage roots from our CNN-based using our **”young” cassava root dataset**.

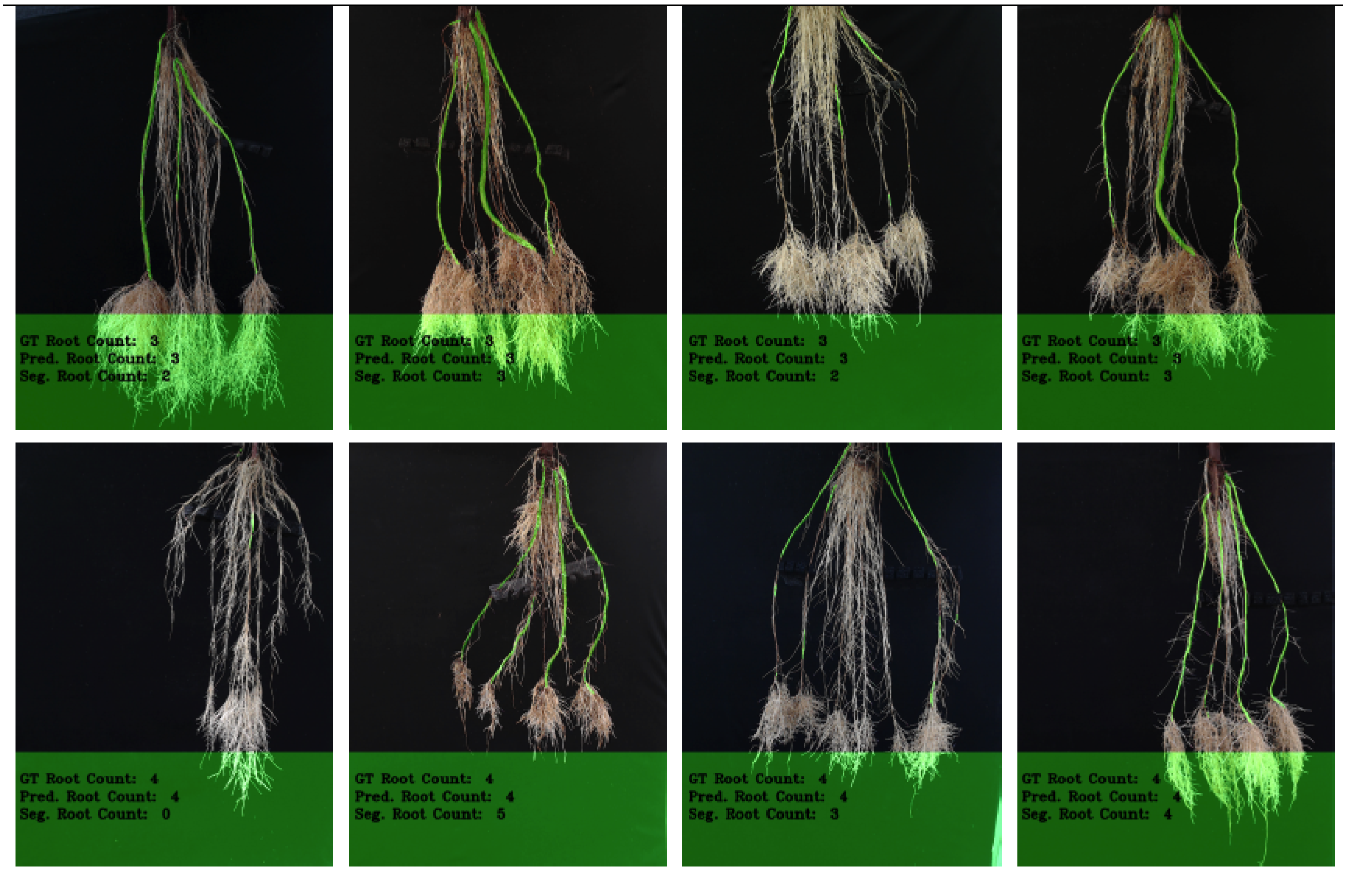

Figure S14: The figure shows a second set of supplementary results of counted storage roots from our CNN-based using our **”young” cassava root dataset**.

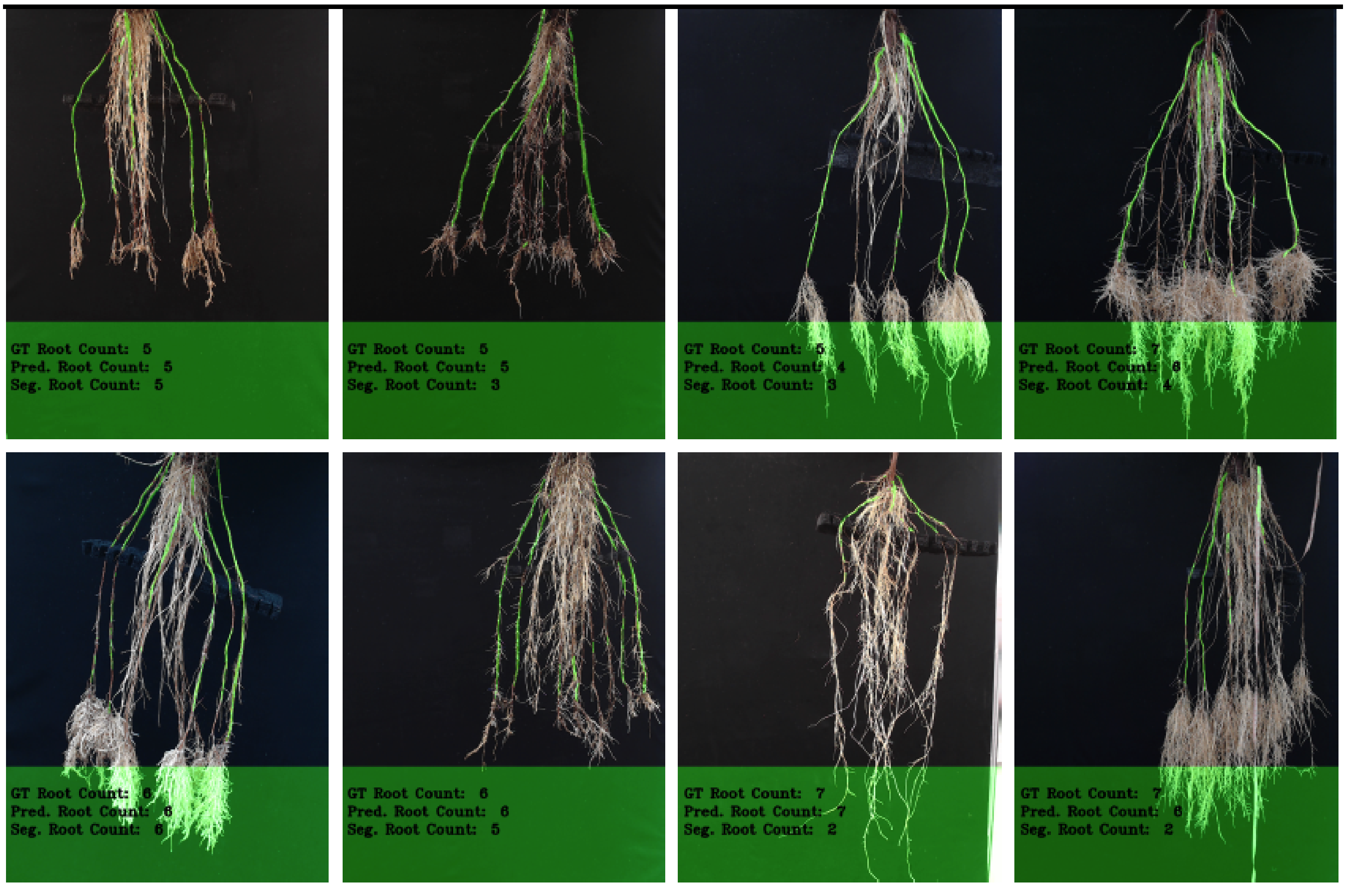

Figure S15: The figure shows a third set of supplementary results of counted storage roots from our CNN-based using our **”young” cassava root dataset**.
